# Tumor necrosis factor-inducible gene 6 protein and its derived peptide ameliorate liver fibrosis by repressing CD44 activation in mice with alcohol-related liver disease

**DOI:** 10.1186/s12929-024-01042-5

**Published:** 2024-05-24

**Authors:** Jinsol Han, Chanbin Lee, Hayeong Jeong, Seunghee Jeon, Myunggyo Lee, Haeseung Lee, Yung Hyun Choi, Youngmi Jung

**Affiliations:** 1 Department of Integrated Biological Science, College of Natural Science, Pusan National University, Pusan, 46241 Republic of Korea; 2 Institute of Systems Biology, College of Natural Science, Pusan National University, Pusan, 46241 Republic of Korea; 3 Department of Biological Sciences, College of Natural Science, Pusan National University, Pusan, 46241 Republic of Korea; 4 Department of Pharmacy, College of Pharmacy and Research Institute for Drug Development, Pusan National University, Pusan, 46241 Republic of Korea; 5 Department of Biochemistry, Dong-Eui University College of Korean Medicine, Pusan, 47227 Republic of Korea

**Keywords:** Alcohol-related liver disease, Liver fibrosis, Tumor necrosis factor-inducible gene 6 protein, Cluster of differentiation 44

## Abstract

**Background:**

Alcohol-related liver disease (ALD) is a major health concern worldwide, but effective therapeutics for ALD are still lacking. Tumor necrosis factor-inducible gene 6 protein (TSG-6), a cytokine released from mesenchymal stem cells, was shown to reduce liver fibrosis and promote successful liver repair in mice with chronically damaged livers. However, the effect of TSG-6 and the mechanism underlying its activity in ALD remain poorly understood.

**Methods:**

To investigate its function in ALD mice with fibrosis, male mice chronically fed an ethanol (EtOH)-containing diet for 9 weeks were treated with TSG-6 (EtOH + TSG-6) or PBS (EtOH + Veh) for an additional 3 weeks.

**Results:**

Severe hepatic injury in EtOH-treated mice was markedly decreased in TSG-6-treated mice fed EtOH. The EtOH + TSG-6 group had less fibrosis than the EtOH + Veh group. Activation of cluster of differentiation 44 (CD44) was reported to promote HSC activation. CD44 and nuclear CD44 intracellular domain (ICD), a CD44 activator which were upregulated in activated HSCs and ALD mice were significantly downregulated in TSG-6-exposed mice fed EtOH. TSG-6 interacted directly with the catalytic site of MMP14, a proteolytic enzyme that cleaves CD44, inhibited CD44 cleavage to CD44ICD, and reduced HSC activation and liver fibrosis in ALD mice. In addition, a novel peptide designed to include a region that binds to the catalytic site of MMP14 suppressed CD44 activation and attenuated alcohol-induced liver injury, including fibrosis, in mice.

**Conclusions:**

These results demonstrate that TSG-6 attenuates alcohol-induced liver damage and fibrosis by blocking CD44 cleavage to CD44ICD and suggest that TSG-6 and TSG-6-mimicking peptide could be used as therapeutics for ALD with fibrosis.

**Supplementary Information:**

The online version contains supplementary material available at 10.1186/s12929-024-01042-5.

## Background

Alcohol-related liver disease (ALD) is a globally prevalent chronic liver disease caused by chronic or binge consumption of alcohol [1]. ALD encompasses a broad spectrum of conditions, including alcoholic fatty liver, alcoholic hepatitis, and alcoholic cirrhosis [2]. In particular, alcoholic hepatitis and alcoholic cirrhosis are related to long-term alcohol abuse and are characterized by disorganized liver architecture with fibrosis [3, 4]. Liver fibrosis is a common scarring response to chronic liver injury and replaces functional liver tissue with excess accumulation of extracellular matrix (ECM), leading to loss of liver function and liver failure [5, 6]. Hepatic stellate cells (HSCs) are key cells involved in the pathogenesis of liver fibrosis [7]. Activated HSCs or myofibroblasts are the main producers of ECM in the liver [8, 9]. Given that liver fibrosis is an essential indicator of liver complications in patients with ALD, regulating HSC activation has been considered an important strategy to prevent ALD progression [10, 11]. However, no drugs regulating HSC activation have been developed, and the currently available treatment for ALD patients with fibrosis is to abstain from drinking or death-delaying treatment until liver transplantation becomes possible [11, 12]. Hence, the development of novel therapeutic agents directly targeting ALD with fibrosis is urgently needed.

Tumor necrosis factor-inducible gene 6 protein (TSG-6), a cytokine released from mesenchymal stem cells (MSCs), has been identified as an anti-inflammatory factor in various organs [13, 14]. In damaged liver, TSG-6 has been shown to attenuate hepatic damage and contribute to a successful repair process. In a nonalcoholic steatohepatitis (NASH)-like animal model, TSG-6 protected hepatocytes from lipotoxicity by increasing autophagy influx in these cells and attenuated hepatic inflammation and fibrosis [15, 16]. The antifibrotic effect of TSG-6 in the liver was directly proven in a CCl_4_-induced liver fibrosis model in which TSG-6 suppressed HSC activation by reprogramming activated HSCs into stem-like cells [17]. These findings indicate that TSG-6 has therapeutic potential for liver fibrosis. However, the detailed mechanism underlying the antifibrotic effect of TSG-6 in the liver remains unclear.

Cluster of differentiation 44 (CD44) is a multifunctional cell-surface receptor that regulates cell adhesion, proliferation, survival, motility, and migration [18, 19]. When activated, CD44 undergoes sequential proteolytic cleavage into an extracellular domain (soluble CD44) and a CD44 intracellular domain (CD44ICD) [20–22]. Then, the cleaved CD44ICD fragment translocates into the nucleus and promotes the transcription of several genes impacting cell migration and CD44 itself [22]. In the liver, CD44 was detected in nonparenchymal cells, such as Kupffer cells, hepatic progenitor cells and HSCs, and among these cells, it was shown to be highly expressed in activated HSCs [23, 24]. Hepatic CD44 expression was reported to be upregulated in the fibrotic livers of patients with various liver diseases, such as viral hepatitis and NASH [24–26]. In addition, soluble CD44 at the serum level was shown to be elevated with the severity of steatosis, and the level of soluble CD44 was higher in NASH patients than in those without NASH [26]. Based on the association of CD44 with liver fibrosis, targeting CD44 is emerging as a potential therapeutic strategy for orchestrating liver fibrosis [24]. However, the effect of CD44 activation via cleavage in liver disease has not been fully elucidated. There is only one study showing the action of CD44ICD in the liver; Yang et al. [27] demonstrate that CD44ICD promotes the transcription of profibrogenic NOTCH1 signaling in HSCs. Wang et al. [28] showed that TSG-6 bound to CD44 in activated HSCs and induced HSC inactivation, but how the interaction of TSG-6 with CD44 induces HSC inactivation remains to be determined.

In the present study, we investigated the inhibitory effect of TSG-6 on liver fibrosis using an animal model of chronic alcohol exposure in which mice were fed an alcohol diet for a long period of time to induce liver fibrosis. TSG-6 treatment attenuated alcohol-induced hepatic damage, including fibrosis. TSG-6 binding to CD44 masked the proteolytic cleavage region of CD44 from matrix metalloproteinase 14 (MMP14), blocked CD44ICD generation in HSCs, and suppressed HSC activation and liver fibrosis in ALD mice. Based on the action of TSG-6 in ALD, the peptide YJ was designed to block MMP14 action by interacting with its catalytic site and exerted antifibrotic effects both in vitro and in vivo. These results suggest that both TSG-6 and the TSG-6-derived peptide YJ have therapeutic potential for ALD with liver fibrosis.

## Materials and methods

### Animal experiments

Male C57BL/6 mice (wild-type; WT) were purchased from Hana Biotech (Gyeonggi-do, Republic of Korea), housed with 12-h light/dark cycle and all mice were provided an adequate acclimation period to allow them to stabilize to their new environment. To determine the time interval for TSG-6 treatment, we analyzed in vivo pharmacokinetic (PK) properties of intraperitoneal injected TSG-6 in acute ALD model (Supplementary Fig. 1a). 7-week-old C57BL/6 male mice were acclimatized for a week and fed either Lieber-DeCarli ethanol liquid diet (710260; Dyets, Bethlehem, PA, USA) containing 5% (v/v) of ethanol or isocaloric Lieber-DeCarli regular control liquid diet (710027; Dyets) for 10 days. On the morning of the 11th day, ethanol-fed mice and pair-fed mice received a single intragastric gavage of ethanol (5 g/kg of body weight) and a single intragastric gavage of isocaloric maltodextrin solution (9 g/kg of body weight), respectively [29]. Nine hours post the gavage feeding, 1.5 μg/kg of human recombinant TSG-6 was intraperitoneally injected to these mice: Pair + TSG-6 (*n* = 5), EtOH + TSG-6 (*n* = 6). Serial blood samples were collected from jugular vein at 10 min, 30 min, 1 h, 2 h, 12 h, 24 h and 48 h after the administration of TSG-6. To induce chronic ALD, 7-week-old C57BL/6 male mice were acclimatized for a week and fed the Lieber-DeCarli ethanol liquid diet (710260; Dyets) containing 5% (v/v) of ethanol for 12 weeks. As a control, mice were fed with an isocaloric Lieber-DeCarli regular control liquid diet (710027; Dyets). At 9 weeks after the liquid diet, mice were divided into randomly eight experimental groups and intraperitoneally injected with 1.5 μg/kg of human recombinant TSG-6 (2104-TS-050; R&D systems, Minneapolis, MN, USA) or 3.3 μg/kg of peptide YJ or saline as vehicle every other day along with feeding liquid diet for 3 additional weeks: Pair + non-treated (*n* = 7), Pair + Veh (*n* = 12), Pair + TSG-6 (*n* = 13), Pair + YJ (*n* = 13), EtOH + non-treated (*n* = 12), EtOH + Veh (*n* = 13), EtOH + TSG-6 (*n* = 13) and EtOH + YJ (*n* = 13). These experimental mice were sacrificed 12 weeks after the diet feeding to collect blood and liver samples. Animal care and surgical procedures were approved by the Pusan National University Institutional Animal Care and Use Committee and carried out in accordance with the provisions of the National Institutes of Health Guide for the Care and Use of Laboratory Animals (Approval Number PNU-2020–2642).

### Isolation of primary HSCs from mice

Mouse primary HSCs were isolated as described previously [30]. Briefly, WT C57BL/6 male mice were anaesthetized with isoflurane to immobilize them in the recumbent position on a treatment table, and the inferior vena cava was cannulated under aseptic conditions. Livers were perfused in situ with EGTA and collagenase (Sigma-Aldrich, St. Louis, MO, USA) to disperse the cells. Primary HSCs were isolated by differential centrifugation on OptiPrep (Sigma-Aldrich) density gradient and located on the upper layer of 11.5% OptiPrep. In the under layer of 11.5% Optiprep, Kupffer cells (KCs) and liver sinusoidal endothelial cells (LSECs) are located. For the separation between LSECs and KCs, mixed suspension of LSECs and KCs were incubated at 37 °C for 15 min because KCs adhere rapidly to the cell culture dishes contrary to LSECs [31]. Some of isolated cells were deposited in a monolayer onto a defined area of a slide by cytospin centrifugation (Cell spin; Hanil Scientific, Gimpo, Republic of Korea) and others were pelleted then stored at -80 °C for western blot analysis.

### Cell experiments

Human pHSCs (purchased from Zen-Bio Inc., NC, USA) were seeded on 10 cm^2^ plates at a density of 2 × 10^6^ cells or on 60 mm plates at a density of 1 × 10^6^ cells or on 35 mm confocal dish at a density of 3 × 10^4^ cells and cultured in DMEM (Gibco) supplemented with 10% FBS (Gibco) and 1% P/S (Gibco) at 37 °C in a humidified atmosphere containing 5% CO_2_. To evaluate effect of TSG-6 or YJ on human pHSCs, pHSCs at 70–80% confluence was serum-starved overnight in medium containing no FBS. Then, pHSCs were treated with 20 ng/ml of human recombinant TSG-6 (2104-TS-050; R&D systems) or 40 ng/ml of YJ or vehicle. The concentration of TSG-6 was determined based on our previous study [17].

### MMP14 siRNA transfection

To knockdown MMP14 expression in HSCs, human pHSCs at 50–60% confluence were serum-starved overnight, cultured in antibiotics-free medium with 2% FBS for 24 h, and then transfected with 25 nM of MMP14 siRNA or scramble (Scr) siRNA using Lipofectamine RNAiMAX (Invitrogen, Life Technologies, CA, USA) for 24 h, as described previously [17, 28]. After washing, TSG-6 or vehicle were given to MMP14-suppressed pHSCs for 12 or 24 h.

### Human samples

Human healthy liver tissues were generous gifts from Anna Mae Diehl and Steve S. Choi (Duke University Medical center), and used as normal controls. Those controls were obtained from residual healthy liver tissues of three donor livers that were utilized for split liver transplantation at Duke University Hospital. And these human liver samples used in this study have been described in previous publication [17, 32].

### Liver histology and immunohistochemistry

To examine hepatic morphology and assess liver fibrosis, liver specimens were fixed 10% neutral buffered formalin, embedded in paraffin, and cut into 4 μm sections. Specimens were deparaffinized, hydrated, and stained as usual method with standard hematoxylin and eosin staining (H&E) and Sirius red staining as previously described [17]. For immunohistochemistry (IHC), sections were incubated for 10 min in 3% hydrogen peroxide to block endogenous peroxidase. Antigen retrieval was performed by heating in 10 mM sodium citrate buffer (pH 6.0) for 10 min or incubating with 0.2% pepsin for 10 min. Sections were blocked in protein blocking solution (X9090; Dako, Carpinteria, CA, USA) for 30 min and incubated with primary antibodies, rabbit anti-Col1a1 (NBP1-30054; Novus Biologicals, LLC, USA), mouse anti-α-SMA (A5228; Sigma-Aldrich), rat anti-F4/80 (ab6640; Abcam, Cambridge, MA, USA) and rabbit anti-CD68 (ab125212; Abcam) or non-immune sera to demonstrate staining specificity at 4 °C overnight. Polymer horseradish peroxidase (HRP) anti-rabbit (K4003; Dako), anti-mouse (K4001; Dako) or HRP anti-rat IgG (A110-105P; BETHYL, Montgomery, Texas, USA) was used as the secondary antibody. 3,3'-Diaminobenzidine (DAB) (K3466; Dako) was employed for the detection procedure.

### Cell proliferation assay

Cell proliferation was measured with a Cell Titer Proliferation Assay (MTS; G3580; Promega) according to the manufacturer’s instructions. In brief, human pHSCs at a density of 5 × 10^3^ cells/well were plated in 96-well plates and treated with either vehicle or peptide YJ for 24 h or 48 h. After treatment, 10 μl of MTS reagent was added to each well, and the plates were incubated in 37 °C in a CO_2_ incubator until the color developed. Absorbance was measured at the wavelength of 490 nm using a Glomax multidetection system (Promega).

### Cell quantification

For quantification analysis of F4/80, or CD68, 10 randomly chosen 40X fields/section were evaluated for each mouse. The positive cells for F4/80, or CD68 were quantified by counting the total number of positive cells/field and dividing by the total number of hepatocytes/field for each mouse. To quantify nuclear CD44ICD, total number of nuclear CD44ICD in pHSCs/field were counted and divided by the total number of pHSCs/field.

### Immunofluorescence staining

For double immunofluorescence staining, cultured human pHSCs on coverslips or cytospun mouse pHSCs were fixed and permeabilized with 4% paraformaldehyde and Triton X-100, respectively. These cells were washed with TBS and blocked in protein blocking solution (X9090; Dako) for 30 min and incubated with first primary antibody, CD44ICD (KAL-KO601; CosmoBio; Carlsbad, CA, USA) or MMP14 (af918; R&D Systems) at 4 °C overnight and followed by Alexa Fluor 568-conjugated goat anti-rabbit IgG (A10042, Invitrogen) or Alexa Fluor 647-conjugated donkey anti-goat IgG (A21447, Invitrogen) for 30 min. For second primary antibodies, sections were incubated with α-SMA (A5228; Sigma-Aldrich) or CD44 (ab157107; Abcam) for 2 h at room temperature and followed by Alexa Fluor 488-conjugated chicken anti-mouse IgG (A21200, Invitrogen) or Alexa Fluor 568-conjugated goat anti-rabbit IgG (A10042, Invitrogen) for 30 min. 4’,6-diamidino-2-phenylindole (DAPI) were employed in the counterstaining procedure. The specimens were then observed and analyzed by confocal microscope DMi8-S (Leica Microsystems, Wetzlar, Germany) or CLSM21 (Carl Zeiss Inc., Thornwood, NY, USA).

### Oil Red O staining

Frozen liver sections were fixed with 4% paraformaldehyde in PBS for 10 min. After fixation, the sections were washed with distilled water three times for 30 s per rinse. Next, sections were incubated with 100% propylene glycol (PEG, Sigma-Aldrich) for 10 min then stained with Oil Red O (0.5% in PEG, Sigma-Aldrich) revealing lipid droplets. Following incubation of staining solution for 10 min, the sections were washed in 60% PEG for 6 min. Nuclei was counterstained with hematoxylin for light microscopic examination.

### Hepatic triglyceride assay

Hepatic triglyceride (TG) levels were measured using Triglyceride Colorimetric assay kit (10010303; Cayman Chemical), following the manufacturers’ instructions. Briefly, liver tissue was homogenized using NP40 and tissue homogenates were centrifuged at 10,000 r.c.f. for 10 min at 4 °C. The supernatants containing TG were used subsequent biochemical analysis. 10 μl of supernatants and 150 μl of enzyme solution were added to each well and then incubated for 15 min. Absorbance was measured at the wavelength of 540 nm using a Glomax multi-detection system (Promega).

### Measurement of AST/ALT

Serum aspartate aminotransferase (AST/GOT, glutamate–oxaloacetate transaminase) and alanine aminotransferase (ALT/GPT, glutamate pyruvate transaminase) were measured using GOT reagents (AM103-K; Asan Pharmaceutical) and GPT reagents (AM102-K; Asan Pharmaceutical) according to the manufacturer's instructions. Briefly, 20 μl of mouse serum was incubated in 100 μl of GOT buffer for 30 min or GPT buffer for 60 min at 37 °C. After incubation, 100 μl of 0.0198% 2,4-dinitrophenylhydrazine colorimetric solution was added to samples, and then these samples were incubated at room temperature for 20 min. After then, 1 ml of 0.4 N NaOH were added and incubated at RT for 10 min. The absorbance was read at 505 nm and results were expressed as International Units Per Liter (IU/L).

### Enzyme-linked immunosorbent assay

To assess in vivo PK properties of human recombinant TSG-6 in ALD mice, levels of human recombinant TSG-6 in mice serum were measured using a human ELISA kit of TSG-6 (EH472RB; Invitrogen) according to the manufacturer’s instructions. Briefly, serum were incubated in plates coated with antibody for overnight at 4 °C with gentle shaking. Biotin conjugates and Streptavidin-HRP were added to each well sequentially. TMB substrate was added and incubated in the dark for 30 min to develop color, and color development was ceased with stop solution. Washing of each well were performed between every incubation step with washing solution. Absorbance was measured at the wavelength of 450 nm using a Glomax multi-detection system (Promega).

### Western blot assay

Total protein was extracted from primary cells or freeze-clamped liver tissue samples that had been stored at − 80 °C. Samples were homogenized in Triton lysis buffer supplemented with protease inhibitor (Complete Mini; Roche, Indianapolis, IN, USA) and centrifuged at 13,000 × g. for 15 min at 4 °C. The supernatants containing protein extracts were used in subsequent biochemical analysis. To separate the nuclear fractions, cells were homogenized and suspended in buffer A (10 mM HEPES, 50 mM NaCl, 1 mM DTT, 0.1 mM EDTA, 0.1 mM PMSF) with protease inhibitors (Roche) and incubated on ice for 20 min. After adding 0.1% NP-40, the cell lysates were incubated on ice for an additional 20 min, then centrifugated at 5000 × r.c.f. for 2 min. The supernatants were saved for cytosolic fraction and the pellets resuspended with buffer B (20 mM HEPES, 400 mM NaCl, 1 mM DTT, 1 mM EDTA, 1 mM PMSF, 1 mM EGTA) and incubated on ice for 30 min. After centrifugation at 13,000 r.p.m for 15 min, the supernatants were collected for the nuclear fraction. The supernatants containing nuclear protein extracts were used in subsequent biochemical analyses. Protein concentration was measured with a Pierce BCA Protein Assay kit (Thermo Scientific, USA). To denature and reduce protein samples, proteins were boiled in 5X sample buffer containing β-mercaptoethanol and sodium dodecyl sulfate (SDS) at 100 °C for 10 min. Total 50 μg of protein lysates was separated by SDS–polyacrylamide gel electrophoresis (PAGE) on 10 or 12% tris–glycine gel and transferred onto a 0.45 μm pore size polyvinylidene difluoride membrane (Millipore, Darmstadt, Germany). Primary antibodies against rabbit anti-TSG-6 (sc-30140; Santa Cruz Biotechnology, Inc., Dallas, TX, USA), rabbit anti-CD44 (ab157107; Abcam), rabbit polyclonal anti-CD44ECD (A12410; Abclonal; Woburn, MA, USA), goat anti-MMP14 (af918; R&D Systems), mouse monoclonal anti-α-SMA (A5228; Sigma-Aldrich), rabbit polyclonal anti-Col1α1 (NBP1-30054; Novus Biologicals), rabbit polyclonal anti-VIMENTIN (sc-5565; Santa Cruz), rabbit polyclonal anti-GFAP (z0334; Dako), rabbit polyclonal anti-TGF-β (3711; Cell Signaling Technology), rabbit polyclonal anti-Cleaved caspase-3 (9661; Cell signaling technology, Danvers, MA, USA), polyclonal anti-Caspase-3 (9662; Cell Signaling), rabbit anti-Lamin B1 (ab16048; Abcam), mouse monoclonal anti-Glyceraldehyde 3-phosphate dehydrogenase (GAPDH; MCA4739; Bio-rad, Oxford, UK) were used in this experiment. HRP-conjugated anti-rabbit or anti-mouse IgG (Enzo Life Sciences, Inc., Farmingdale, NY, USA) was used as secondary antibody. Protein bands were detected using an EzWestLumi ECL solution (ATTO Corporation, Tokyo, Japan) as per the manufacturer’s specifications (ATTO Corporation, Ez-Capture II). Band intensities were calculated using the CS analyzer 4.0 program (Version1.0.3, ATTO).

### Co-Immunoprecipitation assay

Total proteins from vehicle (PBS) or recombinant TSG-6-given pHSCs at 30 min, 1, 2 and 6 h were extracted. To reduce non-specific binding, cell lysates were precleared with protein G Plus/Protein A agarose beads at 4 °C under rotary agitation for 2 h. Sample were centrifuged at 13,000 r.p.m. for 5 min and supernatants were incubated with mouse anti-His tag primary antibody (sc-8036; Santa Cruz) for detecting recombinant TSG-6 or goat anti-MMP14 primary antibody (af918; R&D Systems) at 4 °C overnight. Protein G Plus/Protein A agarose beads (sc-2003; Santa Cruz) were added to each sample and incubated at 4 °C under rotary agitation for 2 h. Sample were centrifuged at 6000 r.p.m. for 1 min and supernatants were discarded. Beads bounded with protein were washed three times with lysis buffer and boiled in 5 × sample buffer for 10 min. Resulting clear supernatants were subjected to sodium dodecyl sulphate–polyacrylamide gel electrophoresis (SDS-PAGE) and the following steps were taken for western blot or LC–MS/MS analysis.

### LC–MS/MS analysis

LC–MS/MS analysis were performed by NICEM (Seoul National University, Republic of Korea). A Thermo Scientific Quadrupole-Orbitrap instrument (Thermo Scientific) equipped with Dionex U 3000 RSLCnano HPLC system was used. Mass spectrometric analyses were performed using a Thermo Scientific Orbitrap Exploris 240 mass spectrometer. Fractions were reconstituted in solvent A (Water/Acetonitrile (98:2 v/v), 0.1% Formic acid) and then injected into LC-nano ESI–MS/MS system. Samples were first trapped on a Acclaim PepMap 100 trap column (100 μm x2cm, nanoViper C18, 5 μm, 100Å, Thermo Scientific, part number 164564) and washed for 6 min with 98% solvent A (water/ACN (98:2 v/v), 0.1% Formic acid at a flow rate of 4 μL/min, and then separated on a PepMap RSLC C18 column (75 μm x 15 cm, nanoViper C18, 3 μm, 100Å, Thermo Scientific, part number ES900) at a flow rate of 300 nL/min. The LC gradient was run at 2% to 8% solvent B over 10 min, then from 8 to 30% over 55 min, followed by 90% solvent B (100% ACN and 0.1% Formic acid) for 4 min, and finally 2% solvent B for 20 min. Xcaliber software version 4.4 was used to collect MS data. The Orbitrap analyzer scanned precursor ions with a mass range of 350–1800 m/z with 60,000 resolution at m/z 200. Mass data are acquired automatically using proteome discoverer 2.5 (Thermo Scientific).

### RNA analysis

Total RNA was extracted from mouse liver tissue or human pHSCs by using Trizol reagent (Invitrogen). The concentration and purity of RNA were determined using nanodrop. Template complementary DNA was synthesized from total RNA using the SuperScript First-strand Synthesis System (Invitrogen) according to manufacturers’ instructions. We performed the real-time qRT-PCR analysis by using Power SYBR Green Master Mix (Applied Biosystem) on the manufacturers’ specifications (QuantStudio 1; Thermo Scientific). All reactions were duplicated, and data were analyzed according to the ΔΔCt method. The expression values were normalized to the levels of human or mouse 40S ribosomal protein 9S mRNA. The sequences of all primers used in this study are summarized in supplementary table S1. All PCR products were directly sequenced for genetic confirmation (Macrogen, Republic of Korea).

### Hydroxyproline assay

Hydroxyproline content of the livers was calculated by the method previously described [30]. Briefly, 50 mg of liver tissue was hydrolyzed in 6 N HCl at 110 °C for 16 h. The hydrolysate was evaporated under vacuum and the sediment was re-dissolved in 1 ml distilled water. Sample were filtered using 0.22 μm filter centrifuge tube (Corning Incorporated, Corning, NY, USA) at 16,873 × g. for 5 min. 0.5 ml of chloramine-T solution, containing 1.41 g of chloramine-T dissolved in 80 ml of acetate–citrate buffer and 20 ml of 50% isopropanol, were added and incubated at room temperature for 20 min. Then, 0.5 ml of Ehrlich’s solution containing 7.5 g of dimethylaminobenzaldehyde dissolved in 13 ml of 60% perchloric acid and 30 ml of isopropanol, was added to the mixture and incubated at 65 °C for 15 min. After cooling at room temperature, the standard and samples were measured by a spectrophotometer at 561 nm. Amount of hydroxyproline in each sample was determined using a regression curve from high purity Trans-4-hydroxy-L-proline (Sigma-Aldrich) as a standard. Total hydroxyproline was calculated based on individual liver weights (mg hydroxyproline/mg liver). Data were expressed as fold changes by comparing with hydroxyproline content of the control group.

### Protein network analysis

To classify the function of TSG-6-interacting proteins detected by LC–MS/MS, Protein Analysis Through Evolutionary Relationships (PANTHER) database version 16.0 (http://www.pantherdb.org) was used as bioinformatics tools in compliance with gene ontology (GO) analysis.

### Protein–protein interaction prediction and computational modelling

Protein–protein interaction (PPI) between TSG-6 and proteins detected by LC–MS/MS was calculated by PSOPIA (https://mizuguchilab.org/PSOPIA/) which is a bioinformatic tool that predicts interaction between pairs of protein given their amino acidic sequences and without prior structural information [33]. Protein sequence of TSG-6 and each protein detected by LC–MS/MS analysis were obtained from protein database of NCBI and these sequences were entered in PSOPIA server (https://mizuguchilab.org/PSOPIA/). PSOPIA scores indicating the probability of interaction were retrieved. The least probability of interaction scores 0, and the maximum possibility of protein–protein interaction scores 1.

For computational modeling, the crystal structures of TSG-6 (PDB ID: 1O7B) and MMP14 (PDB ID: 3MA2) were downloaded from the protein data bank (PDB) and structure of CD44 stem region was generated and validated by following a study which elucidated molecular-level details of the stem regions of CD44 [34]. PyMOL (Version-2.5.2) was used to remove water and atoms. Three-dimensional (3D) structures of peptides derived from TSG-6 were predicted using AlphaFold (v2.1.0) with the parameters –max_template_data = 2020–05-14. The required databases were obtained on December 7, 2021 (UniRef90 v.2021_01, BFD, Uniclust30 v.2018_08, MGnify clusters v.2018_12, and Uniclust30 v.2018_08). For the docking, 3D structure files of each proteins and peptides were submitted at HADDOCK 2.4 (https://wenmr.science.uu.nl/haddock2.4) as protein molecules and protein–protein docking was performed with default settings [35]. The default parameters can be found on the HADDOCK server website (https://wenmr.science.uu.nl/haddock2.4/settings). The 3D structures of the best cluster obtained from the HADDOCK results were visualized using PyMOL.

### Peptide synthesis

Peptides were synthesized by Peptron Inc (Daejeon, Republic of Korea). Each peptide was synthesized using a standard solid-phase peptide synthesis protocol using an ASP48S peptide auto synthesizer and purified by the reverse phase HPLC (Shimadzu Prominence HPLC, Japan) using a ACE 10 C18-300 column (21.2 mm × 250 mm, 10 μm, England). Elution was carried out with a water-acetonitrile linear gradient (10 ~ 75% (v/v) of acetonitrile) containing 0.1% (v/v) Trifluoroacetic acid. Molecular weights of the purified peptide were confirmed using LC/MS (Shimadzu LCMS-2020, Japan) and peptide purities were > 95%.

### Statistical analyses

Results are expressed as mean ± S.E.M. Statistically significant differences between the control and treatment groups or subgroups were analyzed with two-tailed unpaired Student’s t-test, two-way analysis of variance (ANOVA) followed by post hoc Tukey’s test. Differences were considered as significant when *P*-values were < 0.05. Statistical analyses were performed using GraphPad Prism 8 (GraphPad Software Inc.). All experiments were repeated independently at least three times and no data were excluded from the analyses. For all animal experiments, mice were randomly assigned to different experimental groups, and investigators were blinded to the groups during the data collection and analysis.

## Results

Before ALD mice were treated with TSG-6, the PK of TSG-6 was assessed to determine the optimal dosing schedule for TSG-6 (Supplementary Fig. 1a). TSG-6 level in serum reached the mean maximum concentration (C_max_) at 2 h, rapidly declined until 12 h, and gradually decreased until 48 h after TSG-6 injection in both groups (Supplementary Fig. 1b). Based on the PK data of TSG-6 and other studies reporting therapeutic potential of recombinant proteins or peptides in mouse models [36–38], TSG-6 was determined to be given to mice every other day. To confirm chronic ALD model, the pathophysiological liver response to an EtOH diet for 9 weeks was examined in these mice. After confirming liver injury, such as histomorphological damage, excessive lipid accumulation, cell death, collagen infiltration and immune cell recruitment, in these mice (Supplementary Fig. 2), TSG-6 was intraperitoneally injected into these mice for an additional 3 weeks with continuation of the EtOH diet to investigate the hepatoprotective effect of TSG-6 in ALD (Supplementary Fig. 3a). Because no significant effect of vehicle was identified in these experimental mice (Supplementary Fig. 3 and 4), the vehicle-treated mice were used as control mice in comparison with TSG-6-injected mice.

**Fig. 1:**
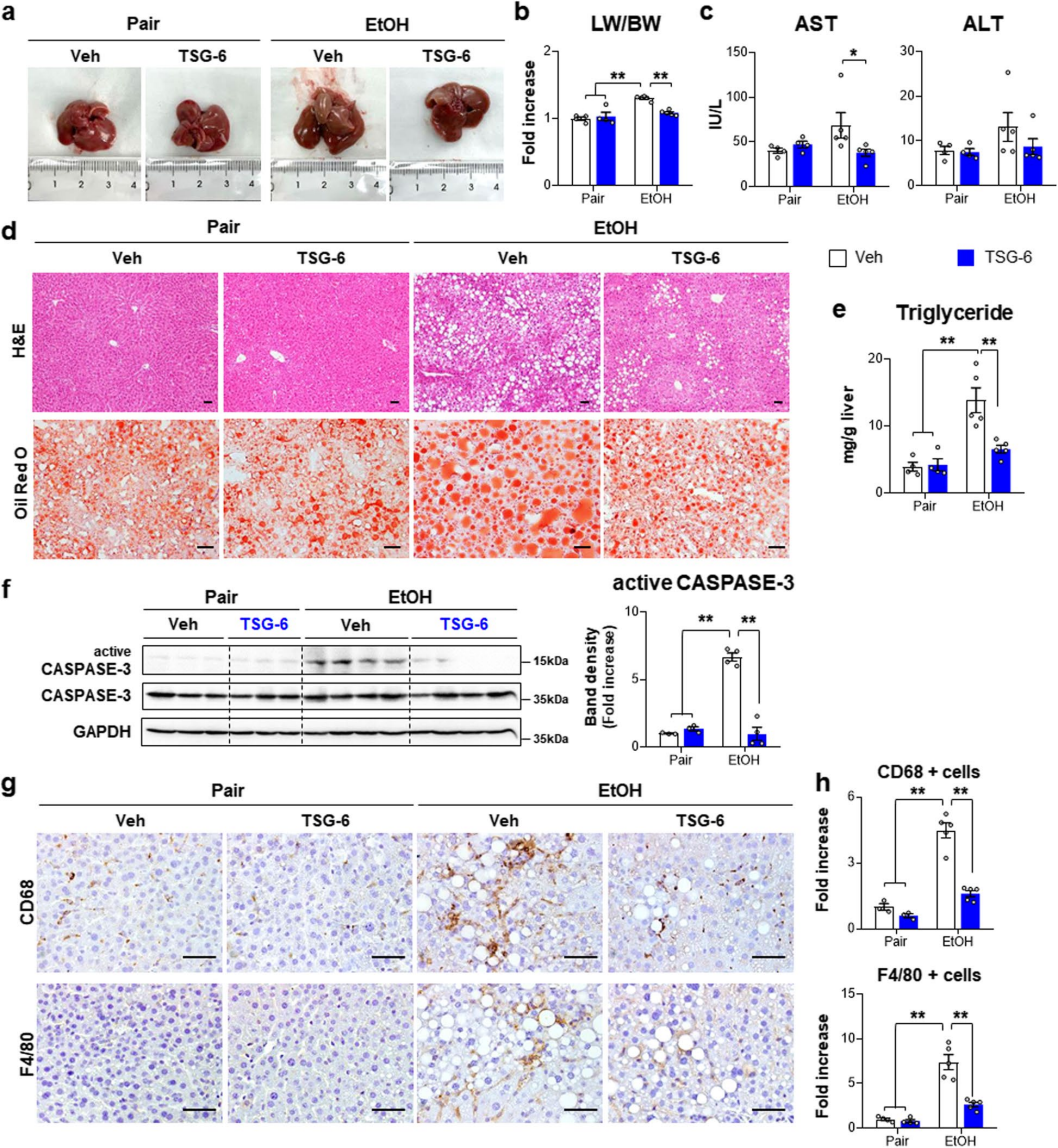
TSG-6 reduces liver injury and inflammation caused by chronic alcohol consumption. **a** Representative macroscopic appearance of livers from vehicle or TSG-6-treated mice fed pair or EtOH diet. **b** The ratio of liver weight to body weight (LW/BW) and **c** the serum levels of aspartate aminotransferase (AST) and alanine aminotransferase (ALT) in these mice. **d** Representative images of hematoxylin and eosin (H&E)-(top panel) and oil-red O-(bottom panel) stained liver sections of mice from each group (Scale bar, 50 μm). **e** Hepatic triglycerides (TG) amount in these mice. **f** Western blot and cumulative densitometric analysis of active CASPASE-3 and total CASPASE-3 in these mice. GAPDH was used as an internal control. Band densities of active CASPASE-3 were normalized to the expression level of total CASPASE-3. **g** Representative images of CD68-(top panel) and F4/80-stained (bottom panel) liver sections from each group (Scale bar, 50 μm). **h** Quantitative CD68- or F4/80-stained data from each group. These data shown represent one of three experiments with similar results from at least four representative mice per each group and are presented as mean ± S.E.M. (*p < 0.05, **p < 0.005). Gray circles represent individual data points

The ethanol diet-fed mice had enlarged and brown-colored livers compared with pair diet-fed mice, while TSG-6 treatment reduced the liver size and weight and restored the liver to a color similar to that in the pair-fed mice (Fig. 1a and b). The AST levels significantly declined from the EtOH + Veh group to the EtOH + TSG-6 group and became similar to those in the pair-fed groups, although ALT levels did not differ among these groups (Fig. 1c). Hematoxylin and eosin (H&E) staining showed severe hepatic injuries, such as the accumulation of macrovesicular lipid droplets, loss of cellular boundaries and ballooned hepatocytes in EtOH diet-fed mice (Fig. 1d top panel). However, these morphological injuries were ameliorated in TSG-6-treated mice exposed to EtOH. Oil red O (ORO) staining presented that the severe accumulation of lipid droplets in the EtOH + Veh group was markedly reduced in the EtOH + TSG-6 group (Fig. 1d bottom panel), and the TG level also supported the reduced lipid accumulation in the EtOH + TSG-6 group (Fig. 1e). In addition, active CASPASE-3, the active form of a key factor in the intrinsic cellular apoptotic cascade, was significantly decreased in the livers of TSG-6-treated EtOH-fed mice compared with the livers of vehicle-given EtOH-fed mice (Fig. 1f). Because abnormal inflammation in the liver is one of the major characteristics during ALD progression, we assessed hepatic inflammation in this mouse model. Immunostaining for F4/80 and CD68, markers of Kupffer cells, showed that these markers-positive cells were less evident in the EtOH + TSG-6 group than in the EtOH + Veh group (Fig. 1g and h).

**Fig. 2:**
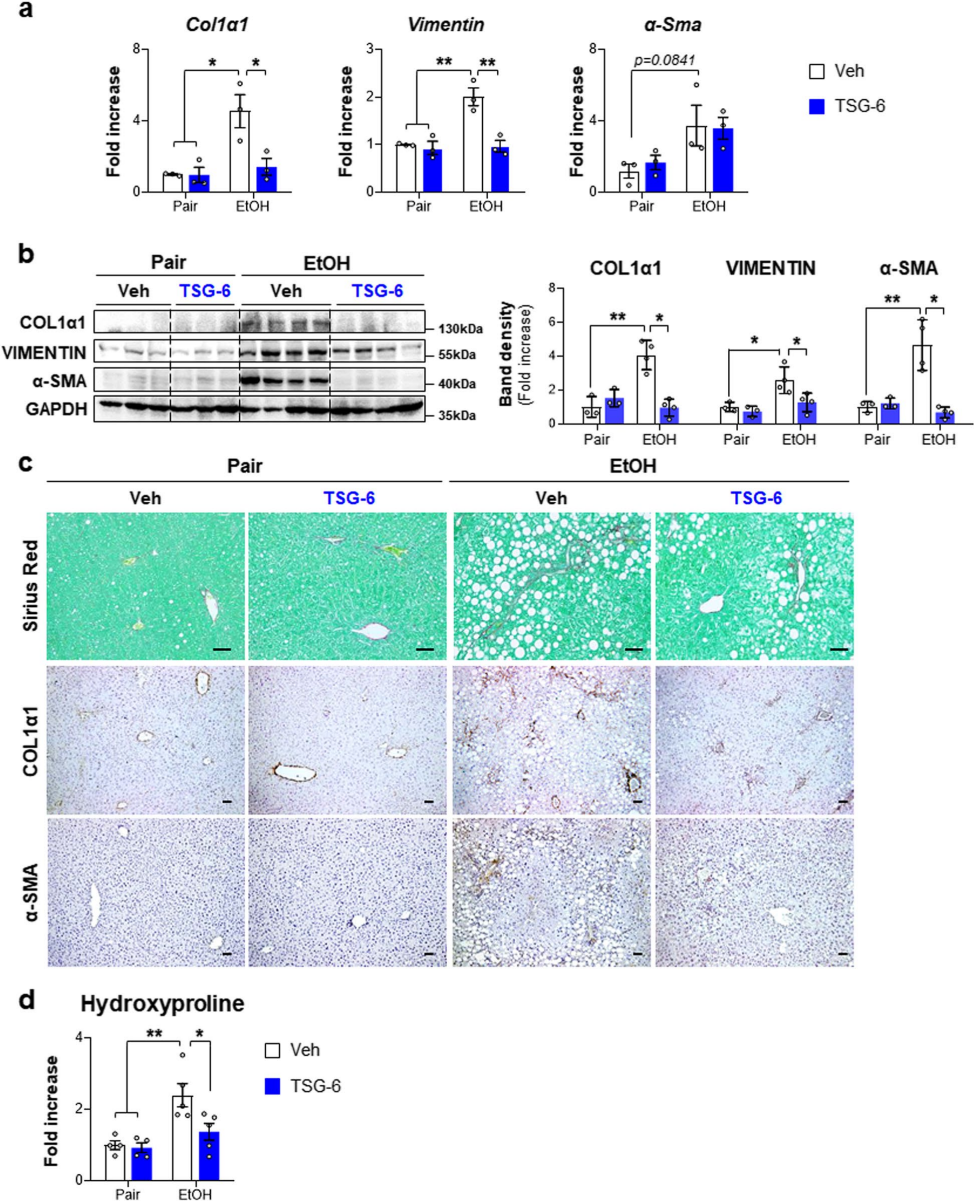
TSG-6 treatment lowers liver fibrosis in EtOH diet-fed mice. **a** qRT-PCR analysis of hepatic *Col1α1*, *Vimentin* and *α-Sma* in representative mice which received pair or EtOH diet with either vehicle or TSG-6. **b** Western blot and cumulative densitometric analysis of COL1α1, VIMENTIN and α-SMA in the liver tissues from representative mice per each group. Band densities were normalized to the expression level of GAPDH, which was used as an internal control. **c** Representative images of Sirius red- (top panel) and COL1α1- (middle panel) and α-SMA-stained (bottom panel) liver sections from these mice (Scale bar, 50 μm). **d** Hepatic hydroxyproline content in the liver tissues from representative mice per each group. These data shown represent one of three experiments with similar results from at least four representative mice per each group and are presented as mean ± S.E.M. (*p < 0.05, **p < 0.005). Gray circles represent individual data points

Chronic EtOH consumption causes massive hepatocyte death and inflammation, eventually leading to liver fibrosis. Because TSG-6 mitigated cell death and inflammation in EtOH-fed mice, fibrotic changes were examined in these mice. The ethanol-fed groups had higher levels of fibrotic markers such as VIMENTIN, Collagen 1 alpha 1 (COL1α1) and α-smooth muscle actin (α-SMA) than pair-fed groups, whereas TSG-6 treatment significantly reduced their expression in EtOH-given mice (Fig. 2a and b). Sirius red staining and immunostaining for COL1α1 and α-SMA clearly showed that the apparent collagen deposition and COL1α1- or α-SMA-positive cells in the EtOH + Veh group were notably reduced in the EtOH + TSG-6 group (Fig. 2c). The fibrotic change was supported by biochemical analysis of hepatic hydroxyproline showing less amount of collagen fibrils in TSG-6-exposed mice than in vehicle-given mice during EtOH feeding (Fig. 2d). Taken together, these results suggest that feeding a chronic ethanol diet causes severe hepatic damage mimicking ALD pathogenesis in humans and that TSG-6 attenuates hepatic injury, especially fibrosis, in an ALD mouse model.

### TSG-6 decreases CD44 activity in HSCs and ethanol-fed mice

**Fig. 3:**
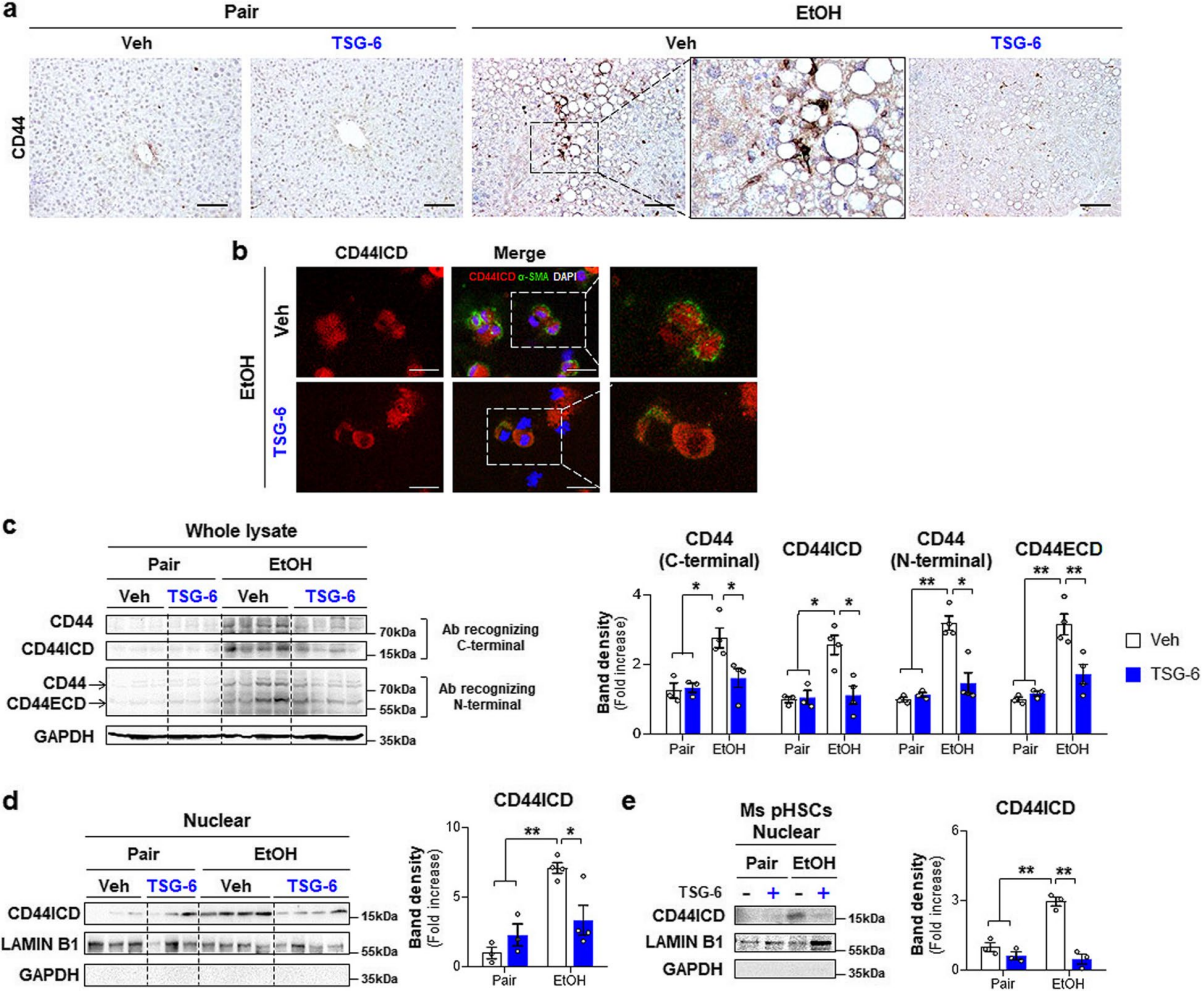
TSG-6 suppresses expression of CD44 and CD44ICD in mice fed EtOH diet. **a** Representative images of CD44-stained liver sections from vehicle or TSG-6-treated mice fed pair or EtOH diet. Magnified image of vehicle-treated EtOH-fed mice are shown at X60 (Scale bar, 50 μm). **b** Representative images of double immunofluorescence staining for CD44ICD (red) and α-SMA (green) in pHSCs isolated from these mice. DAPI (blue) was used as nuclear counterstaining (Scale bar, 25 μm). **c** Western blot and cumulative densitometric analysis of CD44, CD44ICD, and CD44ECD in whole lysate and **d** CD44ICD in nuclear extract from the liver tissues of representative mice from these group. Internal control used GAPDH for whole lysate and LAMIN B1 for nuclear fraction, respectively. Band densities were normalized to the expression level of internal control. GAPDH was used for negative control in nuclear fraction. **e** Western blot and cumulative analysis of CD44ICD in nuclear extract from pHSCs from these mice. These data shown represent one of three experiments with similar results from at least four representative mice per each group, and are presented as the ± S.E.M. (*p < 0.05, **p < 0.005). Gray circles represent individual data points

**Fig. 4:**
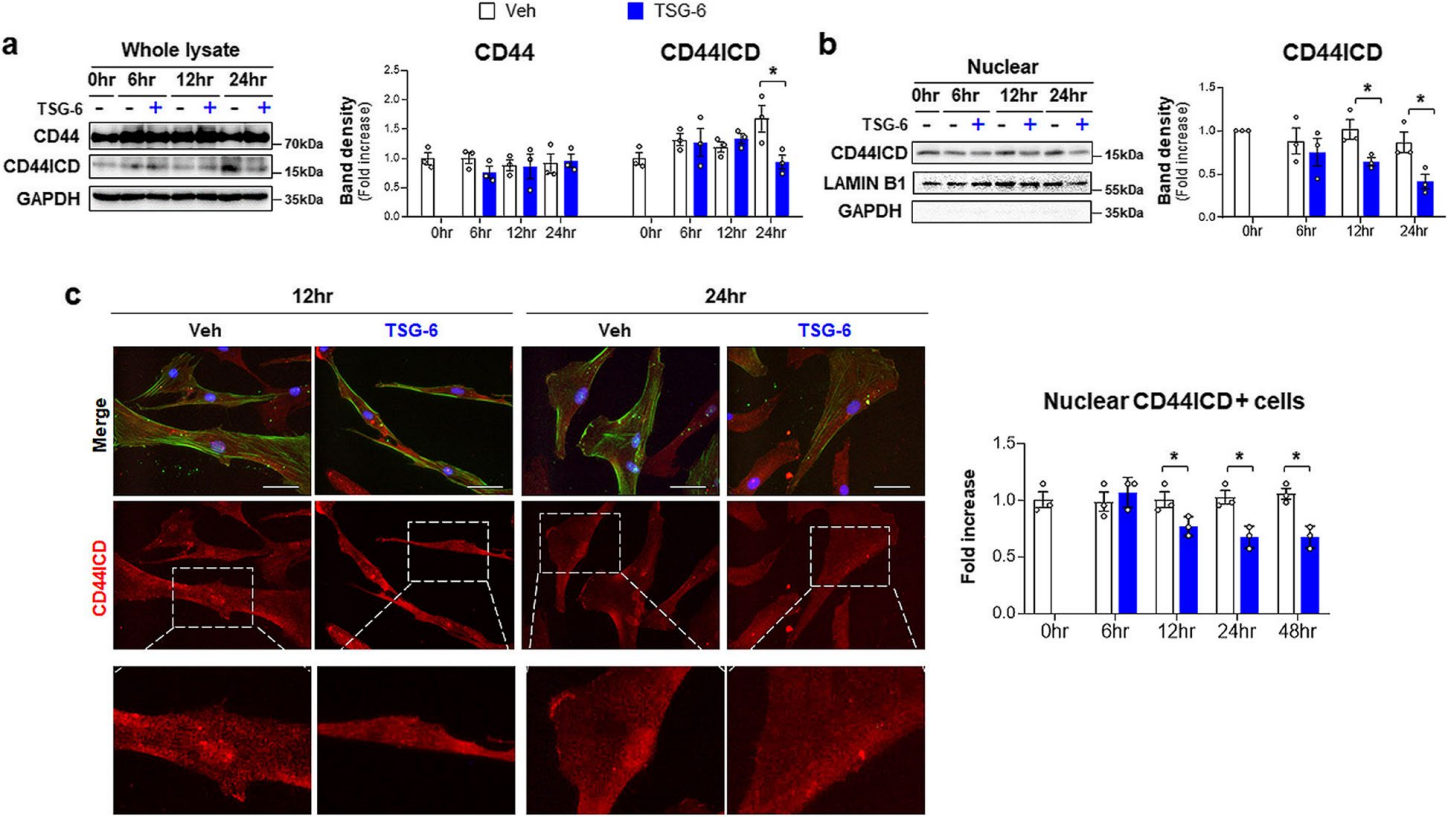
TSG-6 abrogates production and nuclear localization of CD44ICD in human primary HSCs. **a** Western blot and cumulative densitometric analysis of CD44 and CD44ICD in whole lysate and **b** CD44ICD in nuclear extract of TSG-6-treated human pHSCs. Internal control used GAPDH for whole lysate and LAMIN B1 for nuclear fraction, respectively. Band densities were normalized to the expression level of internal control. GAPDH was used for negative control in nuclear fraction. (**c**) Representative images of double immunofluorescence staining for CD44ICD (red) and α-SMA (green) and quantitative analysis of nuclear CD44ICD-positive cells. Magnified images of CD44ICD-stained images in bottom panel are shown at X60. DAPI (blue) was used as nuclear counterstaining (Scale bar, 50 μm). These data shown represent one of three experiments with similar results from at least three representative trials and are presented as mean ± S.E.M. (*p < 0.05, **p < 0.005). Gray circles represent individual data points

CD44 cleavage to CD44ICD leads to HSC activation and liver fibrosis [27]. Given that TSG-6 bound with CD44 to inactivate HSCs [28], we examined whether TSG-6 influenced CD44 activation to reduce liver fibrosis in EtOH-fed mice. Immunostaining for CD44 showed that the more apparent accumulation of CD44-positive cells in the livers of ethanol-fed mice than pair-fed mice was reduced in the livers of ethanol-fed mice with TSG-6. And CD44 expression in the EtOH + Veh group was detected mainly HSC-like cells (Fig. 3a). These findings were confirmed by double immunofluorescent staining showing that HSCs isolated from EtOH-fed mice had nuclear CD44ICD (Fig. 3b). The expression of full-length CD44 and CD44ICD, the cleaved intracellular form of CD44, was upregulated in ethanol-fed mice compared with pair-fed mice, but TSG-6 treatment remarkably downregulated their levels in mice fed EtOH (Fig. 3c). In addition, extracellular domain of CD44 (CD44ECD) is known to increase in response to liver damage [24, 26, 39]. EtOH upregulated CD44ECD, but TSG-6 treatment significantly downregulated it in EtOH-treated mice. The analysis of the nuclear expression of CD44ICD revealed that the EtOH diet significantly increased its expression in the liver compared with the pair diet (Fig. 3d). However, TSG-6 injection significantly decreased its nuclear expression in EtOH-treated mice compared with vehicle-treated mice fed EtOH. HSCs isolated from TSG-6-treated EtOH-fed mice also had significantly lower levels of nuclear CD44ICD than those from vehicle-treated EtOH-fed mice (Fig. 3b and e). To further assess whether TSG-6 inhibited CD44 activity in HSCs by blocking CD44ICD expression, human primary HSCs (pHSCs) were treated with TSG-6 for 6, 12, 24 and 48 h. Although full-length CD44 expression was not altered by TSG-6 treatment, the level of CD44ICD was apparently downregulated at 24 h after TSG-6 treatment compared with vehicle treatment in pHSCs (Fig. 4a). Nuclear expression of CD44ICD clearly showed that TSG-6-treated pHSCs had significantly lower levels of nuclear CD44ICD at 12 and 24 h than vehicle-treated pHSCs (Fig. 4b). In addition, double immunofluorescence staining of α-SMA (green) and CD44ICD (red) showed that strong nuclear localization of CD44ICD was observed in vehicle-treated cells at 12 and 24 h, and α-SMA staining was evident in these cells (Fig. 4c and Supplementary Fig. 5). However, HSCs having nuclear localization of CD44ICD was relatively a few at 12 h and rarely detected at 24 and 48 h in TSG-6-treated cells, and α-SMA expression was also reduced at 48 h in these cells. Taken together, these data demonstrate that TSG-6 suppresses CD44 activation by inhibiting CD44ICD expression in HSCs and the livers of ALD mice.

### TSG-6 interacts with MMP14 and reduces its expression in vivo and in vitro

**Fig. 5:**
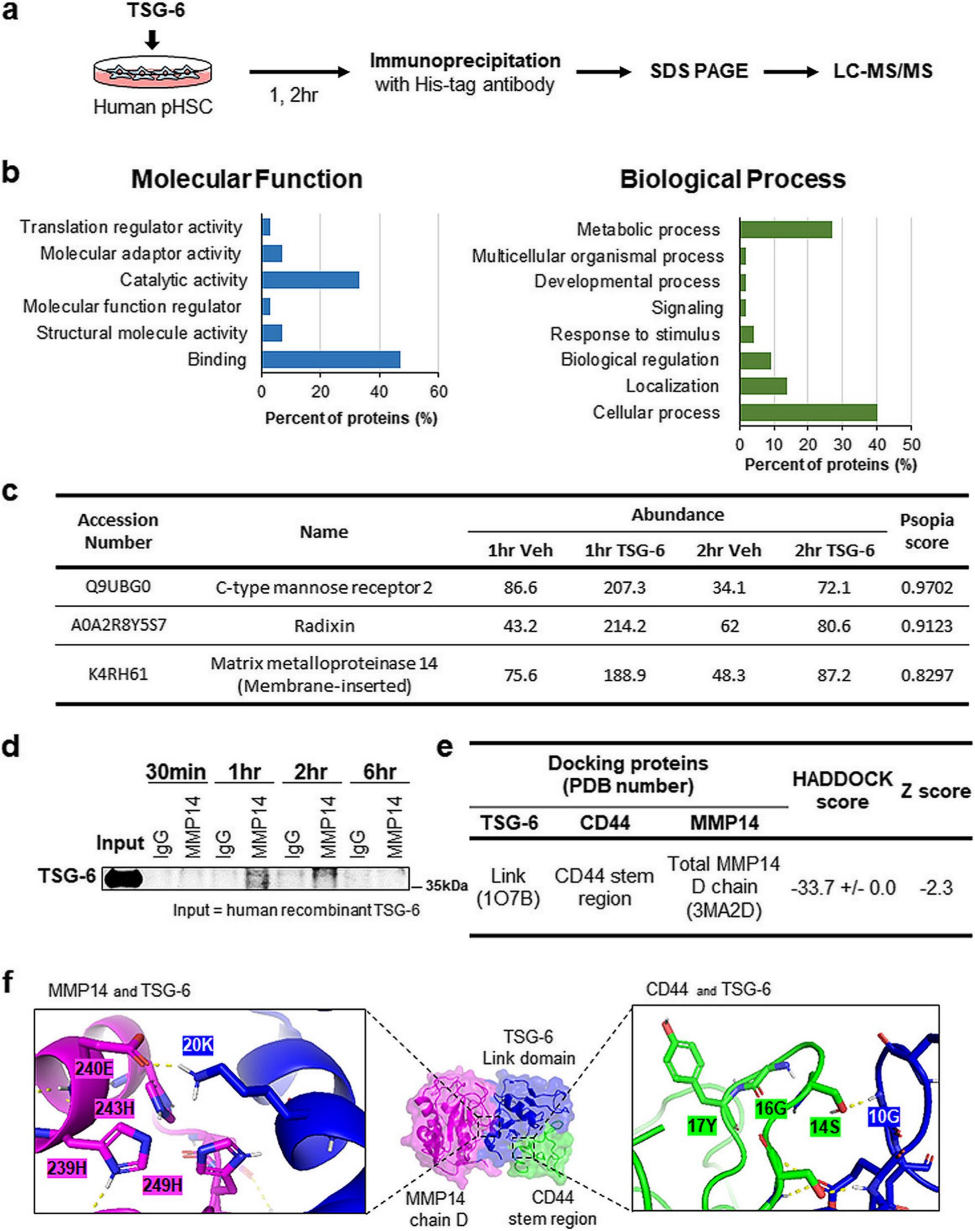
TSG-6 binds with MMP14 in human pHSCs. **a** A scheme for the experimental procedure of co-immunoprecipitation (co-IP) coupled with liquid chromatography-mass spectrometry (LC–MS/MS). **b** Gene ontology enrichment analysis of TSG-6 interaction proteins identified by LC–MS/MS in categories of molecular function and biological process. **c** Top 3 proteins identified by LC–MS/MS having the highest Psopia score and the relative abundance of these proteins. **d** Western blots for TSG-6 in the immunoprecipitated pHSC lysates with MMP14. pHSCs treated with TSG-6 for 30 min, 1, 2, or 6 h were immunoprecipitated with MMP14 antibody or IgG as a negative control. Input sample containing the same amount of TSG-6 protein used for in vitro treatment was employed as a positive control. Data shown represent one of three experiments with similar results. **e** Results of molecular docking (HADDOCK) analysis of TSG-6, CD44 and MMP14. Structures of TSG-6 and MMP14 were obtained from Protein Data Bank (PDB) and structure of CD44 stem region was synthesized according to its sequence. **f** 3D structure of the most stable clusters of TSG-6 (blue), CD44 (green) and MMP14 (magenta) predicted by HADDOCK. Magnified image shows the interacting site of TSG-6 with catalytic region of MMP14 (left panel) and with cleavage site of CD44 (right panel)

To investigate how TSG-6 disturbed CD44 activity to suppress HSC activation, interacting partners of TSG-6 were analyzed by coimmunoprecipitation (co-IP) coupled with liquid chromatography tandem mass spectrometry (LC–MS/MS) (Fig. 5a). Gene Ontology (GO) annotation of the proteins identified by LC‒MS/MS showed that most TSG-6-interacting proteins seemed to be related to binding and catalytic activity in the molecular function category and to regulators of cellular and metabolic processes in the biological process category (Fig. 5b). We selected three proteins that were candidate interaction partners of TSG-6 based on their higher abundance level in TSG-6-exposed pHSCs than in vehicle-given cells and a high PSOPIA score, indicating the possibility of interaction with TSG-6 (Fig. 5c). PSOPIA was assessed by an online protein‒protein interaction prediction server. Among these proteins, MMP14, a membrane-anchored zinc-binding endopeptidase, was chosen for further investigation because it is a well-known key enzyme for CD44 proteolytic cleavage [40–42]. MMP14 is also a marker of HSC activation [43–45]. To confirm the data obtained from LC‒MS/MS analysis, the direct interaction of TSG-6 and MMP14 was examined by co-IP assay. In anti-MMP14 immunoprecipitates from TSG-6-treated human pHSCs, TSG-6 was detected at 1 h, its expression declined at 2 h, and it was rarely observed at 6 h after TSG-6 treatment (Fig. 5d).

To prove the relationship between the interaction of TSG-6 with MMP14 and decreased CD44 activity, MMP14-expressing cells were first analyzed. MMP14 was rarely present in primary hepatocytes, Kupffer cells, and LSECs isolated from healthy livers of mice, whereas its expression was detected in HSCs and was more evident in activated HSCs on Day 7 than HSCs on Day 0 after isolation. MMP14 was also detected in human pHSCs and LX2, HSC line, but not in healthy liver tissue (Supplementary Fig. 6a). Interestingly, MMP14 expression increased in parallel with CD44 expression during HSC activation. Immunofluorescence staining for CD44 (red) and MMP14 (pink) showed their colocalization in the membranes of activated pHSCs from humans and mice, as examined by confocal microscopy (Supplementary Fig. 6b). In the ALD mouse model, MMP14 at both the RNA and protein levels was upregulated with increased CD44 activity and liver fibrosis (Fig. 3, Supplementary Fig. 6c and 6d). However, TSG-6 treatment notably downregulated MMP14 at both the RNA and protein levels, similar to the effect of decreasing CD44 activity and liver fibrosis in the EtOH-treated mice. In line with the in vivo data, the level of MMP14 was significantly lower in TSG-6-given human pHSCs than in vehicle-treated cells at 48 h (Supplementary Fig. 6e). MMP14 expression in these cells rarely changed until 48 h after TSG-6 treatment. Therefore, these data revealed that TSG-6 interacted with MMP14 and reduced its expression in HSCs and ALD mice, as TSG-6 decreased CD44 activation, suggesting that TSG-6 may interrupt MMP14-mediated cleavage of CD44 to inactivate HSCs.

### TSG-6 blocks the catalytic domain of MMP14 and inhibits the cleavage of CD44 into CD44ICD

**Fig. 6:**
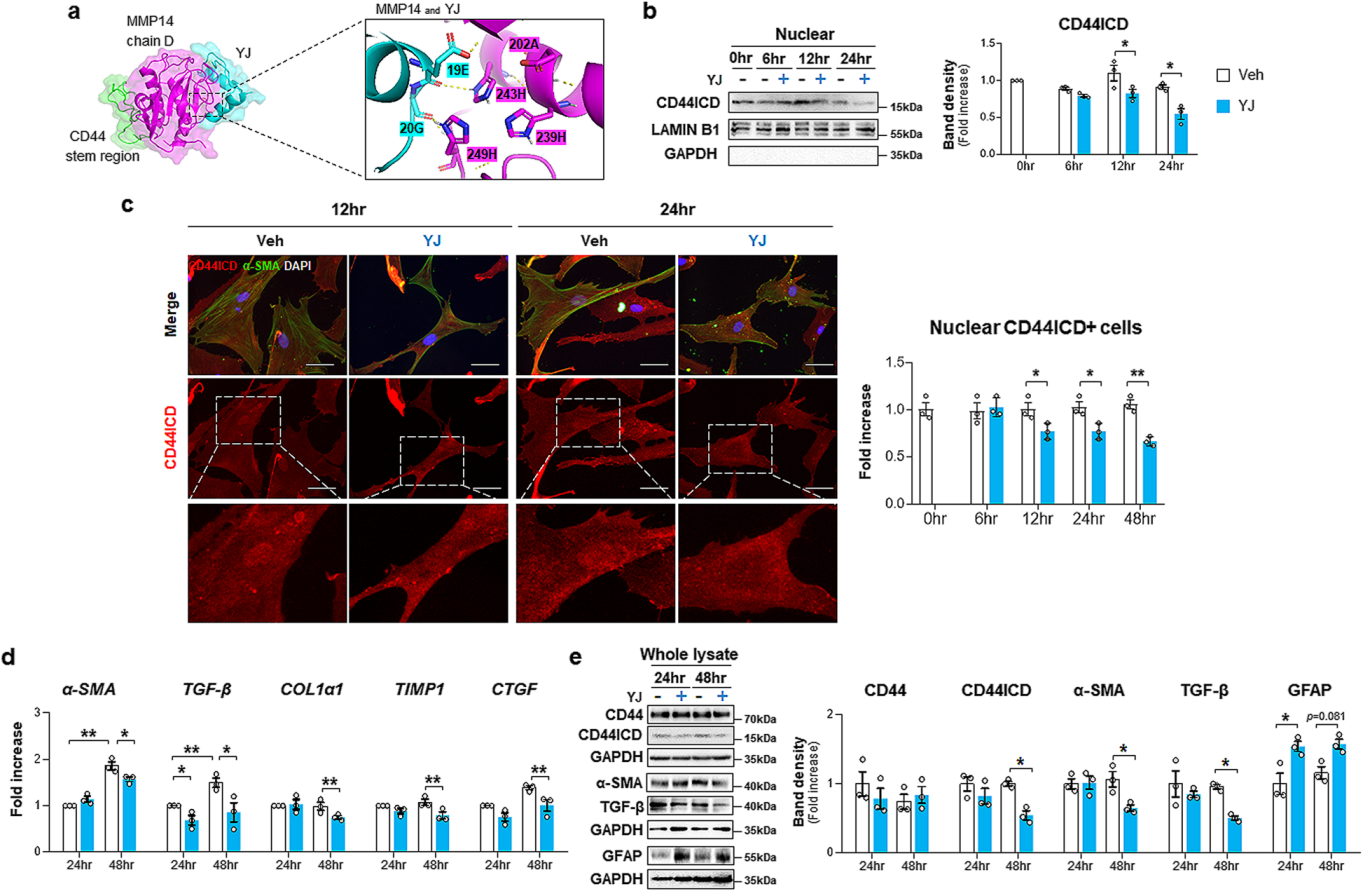
Peptide YJ derived from TSG-6 lessens production and activation of CD44ICD and HSC activation. **a** 3D structure of the most stable clusters of peptide YJ (cyan), CD44 (green) and MMP14 (magenta) predicted by HADDOCK. Magnified image in right panel presents the interaction site of peptide YJ with catalytic region of MMP14. **b** Western blot and cumulative densitometric analysis of nuclear CD44ICD in pHSCs treated with YJ. Band densities were normalized to the expression level of LAMIN B1, which was used as an internal control. GAPDH was used for negative control in nuclear fraction. **c** Representative images of double immunofluorescence staining for CD44ICD (red) and α-SMA (green), and quantitative analysis of nuclear CD44ICD-positive cells. Magnified images of CD44ICD-stained images in bottom panel are shown at X60. DAPI (blue) was used as nuclear counterstaining (Scale bar, 50 μm). **d** qRT-PCR analysis of fibrotic markers including *α-SMA*, *transforming growth factor-β (TGF-β), COL1α1,tissue inhibitor of metalloproteinase 1 (TIMP1)* and *connective tissue growth factor* (*CTGF)* in these cells. **e** Western blot and cumulative analysis for CD44, CD44ICD, TGF-β, α-SMA and glial fibrillary acidic protein (GFAP) in whole lysate of these cells. Band densities were normalized to the expression level of GAPDH, which was used as an internal control. The data shown represent one of three experiments with similar results and are presented as mean ± S.E.M. (*p < 0.05, **p < 0.005). Gray circles represent individual data points

To prove the inhibitory action of TSG-6 in CD44 cleavage induced by MMP-14, we employed computational analysis to predict the structure of the interaction among TSG-6, CD44 and MMP14. Computational modeling of the CD44, MMP14 and TSG-6 complexes was performed by the High Ambiguity Driven protein‒protein DOCKing (HADDOCK; https://wenmr.science.uu.nl/haddock2.4/) web server and analyzed by PyMOL (Schrödinger, Inc.). The most stable cluster with the most negative HADDOCK score and Z score had a structure in which the TSG-6 link domain was located between MMP14 and CD44 (Fig. 5e and f). Specifically, TSG-6 made direct polar contact with conserved histidines, which ligated the active site of the MMP14 catalytic domain (Fig. 5f, magnified left panel). Hence, it is possible that TSG-6 physically inhibits the catalytic activity of MMP14 and protects CD44 cleavage to CD44ICD from MMP14 to reduce HSC activation and liver fibrosis in mice with ALD. Hence, we designed 6 novel peptides with the specific TSG-6 sequence that inhibits the catalytic activity of MMP14 to prove this hypothesis (Supplementary Fig. 7a and b). Using the 3D structures of each peptide, CD44, and MMP14, the potential binding relationships among them were analyzed by HADDOCK. Among these peptides, peptide number 4 formed the most stable complex with a -60.7 ± 22.2 HADDOCK score and -1.2 Z score (Supplementary Fig. 7c). Analysis of the predicted 3D structure of peptide number 4 (cyan) interacting with CD44 (green) and MMP14 (magenta) showed that this peptide bound to the catalytic site of MMP14, similar to TSG-6 (Fig. 6a). After synthesizing peptide #4, named YJ, the optimal concentration of YJ for application and its effect on human pHSCs were first studied. Peptide YJ at the tested concentrations had little impact on cell viability, as assessed by MTS assay (Supplementary Fig. 7d). And, 20 or 40 ng/ml of YJ was given to pHSCs for 24 and 48 h. The activation markers α-SMA and TGF-β were downregulated, and the inactivation marker GFAP was upregulated in cells treated with 40 ng/ml YJ for 24 or 48 h (Supplementary Fig. 7e). Based on the data, 40 ng/ml was determined to be the optimal concentration of YJ for in vitro functional studies.

Peptide YJ-treated human pHSCs showed a significant decrease in CD44ICD in the nucleus after 12 h compared to vehicle-treated cells (Fig. 6b). Double immunofluorescence staining for CD44ICD (red) and α-SMA (green) also supported the significant reduction of nuclear CD44ICD at 12, 24, and 48 h with α-SMA decrease at 24 and 48 h in YJ-given cells compared with vehicle-treated cells (Fig. 6c and Supplementary Fig. 8). In line with the absence of CD44ICD in the nucleus in YJ-exposed pHSCs, peptide YJ significantly alleviated the levels of fibrotic markers such as *α-SMA*, *TGF-β*, *COL1α1*, *TIMP1* and *CTGF* compared with those in vehicle-treated pHSCs at 24 or 48 h (Fig. 6d). Western blotting also confirmed the inhibitory effect of peptide YJ on CD44ICD expression and HSC activation by showing a decrease in CD44ICD, α-SMA and TGF-β and an increase in GFAP (Fig. 6e).

In addition, to confirm MMP14 blockage by TSG-6 contributed to the maintaining intact CD44, MMP14 was suppressed by MMP14-siRNA in activated HSCs. In Scr RNA-treated cells, TSG-6 significantly downregulated CD44ICD. However, MMP14 knockdown rarely impact CD44ICD level in these cells regardless of TSG-6 treatment (Supplementary Fig. 9). Taken together, these findings suggest that TSG-6 suppresses CD44 activity by abrogating the catalytic action of MMP14 on CD44 and contributes to HSC inactivation.

### The TSG-6-based peptide ameliorates alcohol-induced liver damage

**Fig. 7:**
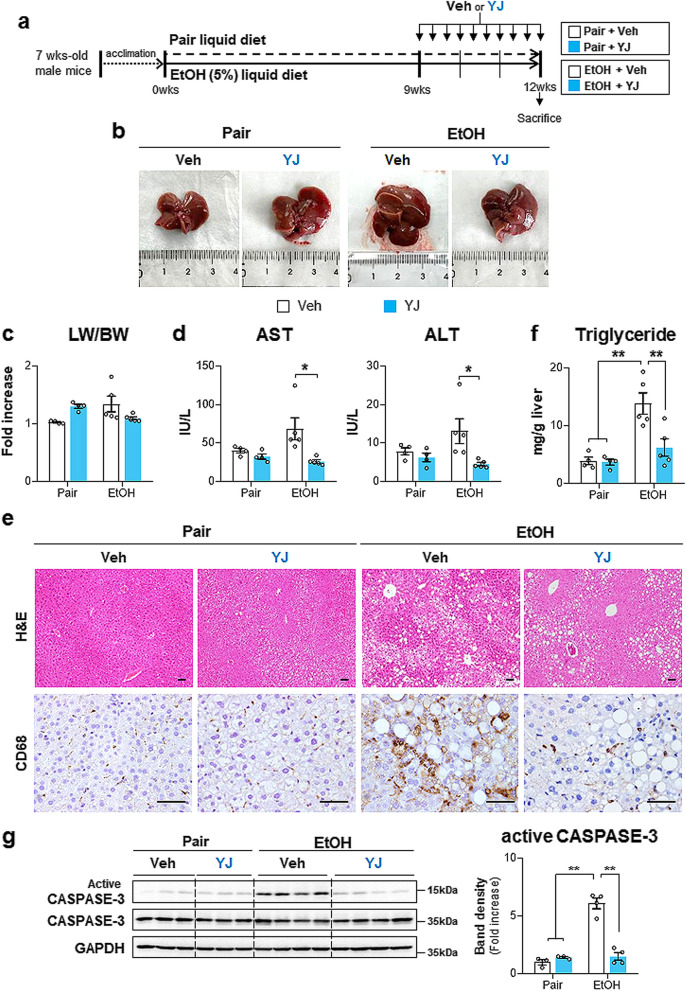
Peptide YJ reduces chronic liver injury caused by EtOH diet. **a** A scheme for animal experiment. 7-week-old male C57BL/6 mice which were acclimatized for a week were fed either isocaloric pair diet or 5% EtOH diet for 9 weeks and additionally treated with the diet for 3 weeks in parallel with i.p injection of either vehicle or peptide YJ. **b** Representative macroscopic appearance of livers from these mice. **c** The ratio of liver weight to body weight, **d** the serum AST and ALT levels in these mice. **e** Representative images of H&E-(first panel) and CD68-(bottom panel) stained liver sections from each group (scale bar, 50 μm). **f** Hepatic TG amount of these experimental mice from each group. **g** Western blot and cumulative densitometric analyses of active CASPASE-3 and total CASPASE-3 in these mice. GADPH were used as internal control. Band densities of active CASPASE-3 were normalized to the expression level of total CASPASE-3. These data shown represent one of three experiments with similar results from at least four representative mice per each group, and are presented as the mean ± S.E.M. (*p < 0.05, **p < 0.005). Gray circles represent individual data points

To examine the physiological function of peptide YJ in the liver, YJ was administered to WT male mice that were chronically fed either ethanol- or pair-containing diets, similar to the TSG-6-treated ALD model (Fig. 7a). Because YJ showed a significant in vitro effect at a concentration twice as high as that of TSG-6 (Supplementary Fig. 7e), it was used at twice the concentration of TSG-6 in vivo. The enlarged liver and increased LW/BW of EtOH-fed mice (EtOH + Veh group) decreased in peptide YJ-treated mice fed ethanol (EtOH + YJ group) (Fig. 7b and c). The levels of serum AST and ALT, morphological damage, and lipid accumulation were ameliorated in the EtOH + YJ group relative to the EtOH + Veh group (Fig. 7d and e top panel). Hepatic TG levels supported that the EtOH + YJ group exhibited less lipid deposition than the EtOH + Veh group (Fig. 7f). In addition, the enhanced inflammation and apoptosis in EtOH-injured livers were significantly decreased by peptide YJ treatment (Fig. 7e bottom panel and g). No significant effect of peptide YJ on pair-fed mice compared to the pair-fed mice treated with vehicle was identified (Fig. 7).

### The peptide YJ mimicking TSG-6 inhibits CD44 activation and mitigates liver fibrosis in mice with ALD

**Fig. 8:**
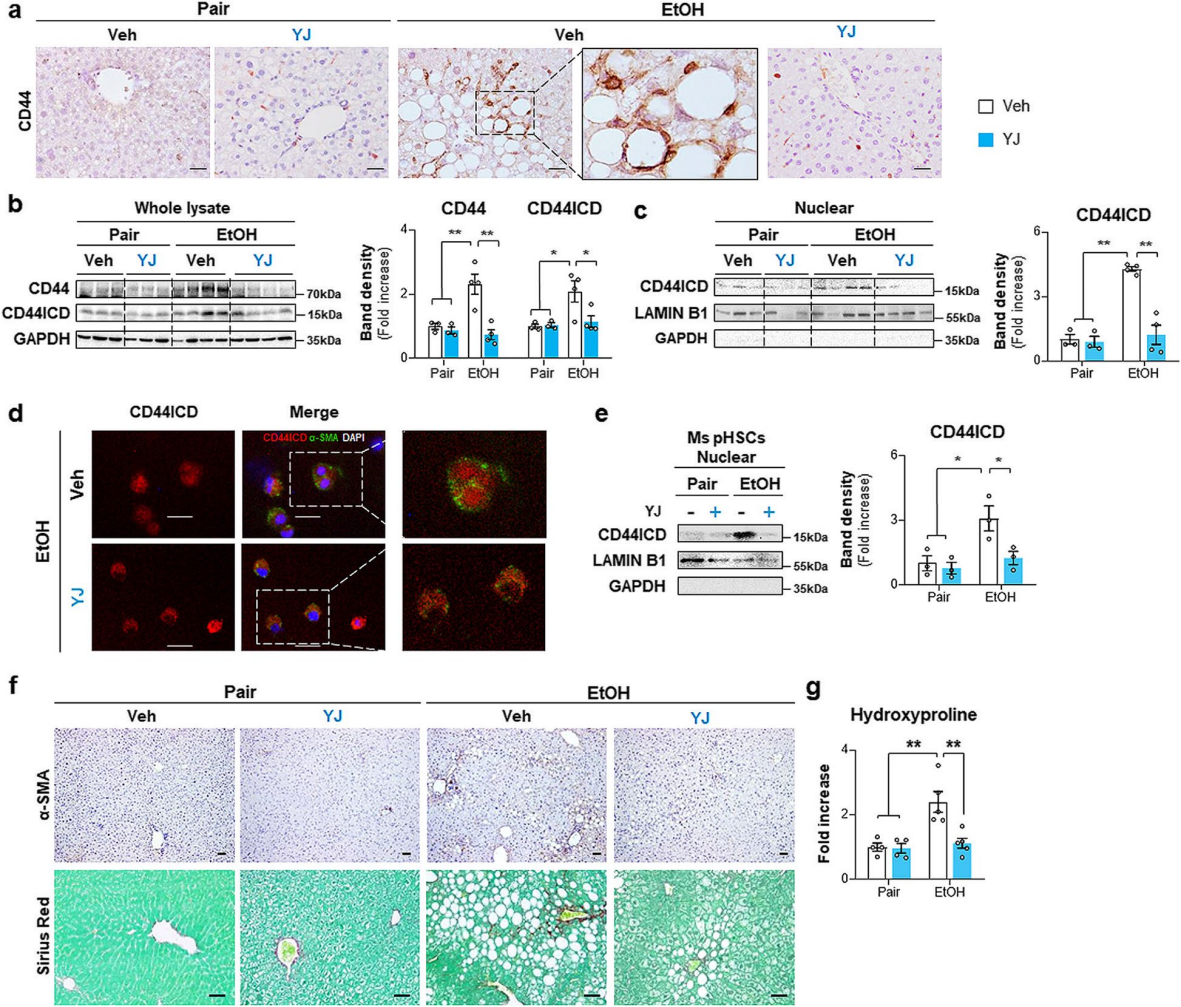
Peptide YJ blocks CD44 cleavage into CD44ICD, and decreases hepatic fibrosis in EtOH-fed mice. **a** Representative images of CD44-stained liver sections from vehicle or peptide YJ-treated mice fed pair or EtOH diet (scale bar, 50 μm). Magnified liver image of EtOH-fed mice treated with vehicle is shown at X60. **b** Western blot and cumulative analysis of CD44 and CD44ICD in whole lysate and **c** CD44ICD in nuclear extract from the liver tissues of representative mice from these group. Internal control used GAPDH for whole lysate and LAMIN B1 for nuclear fraction, respectively. Band densities were normalized to the expression level of internal control. GAPDH was used for negative control in nuclear fraction. **d** Representative images of double immunofluorescence staining for CD44ICD (red) and α-SMA (green) in pHSCs isolated from these mice. DAPI (blue) was used as nuclear counterstaining (Scale bar, 25 μm) **e** Western blot and cumulative analysis of CD44ICD in nuclear extract from these cells. **f** Representative images of Sirius red- and α-SMA-stained liver sections from these mice (scale bar, 50 μm). **g** Hepatic hydroxyproline content in liver tissues from representative mice per each group. These data shown represent one of three experiments with similar results from at least four representative mice per each group and are presented as the mean ± S.E.M. (*p < 0.05, **p < 0.005). Gray circles represent individual data points

To investigate whether the peptide YJ-interacting catalytic region of MMP14 impacted the reduction in CD44ICD and attenuated liver fibrosis in EtOH-fed mice, CD44 expression in these mice was first examined. Treatment with peptide YJ lowered the number of CD44-expressing cells in EtOH-fed mice compared with that in EtOH-treated mice without YJ (Fig. 8a). CD44 and CD44ICD, which were upregulated in whole liver tissues of the EtOH + Veh group, were significantly downregulated in the liver of the EtOH + YJ group, as assessed by western blot (Fig. 8b). Reduced CD44 activity caused by peptide YJ was confirmed by the observation of a significant decrease in nuclear CD44ICD in the EtOH + YJ group compared with the EtOH + Veh group (Fig. 8c). HSCs isolated from EtOH-fed mice also showed apparent nuclear CD44ICD compared with cells from YJ-given mice administered EtOH (Fig. 8d and e).

In EtOH-fed mice, the expression of VIMENTIN and COL1α1 at both the RNA and protein levels was lower in YJ-treated mice than in vehicle-treated mice (Supplementary Fig. 10). Although the level of *α-Sma* RNA rarely showed a significant difference among the groups, the amount of α-SMA protein and the accumulation of α-SMA-expressing cells were notably alleviated in the EtOH + YJ group compared with the EtOH + Veh group (Supplementary Fig. 10a and b and Fig. 8f top panel). In line with these findings, Sirius red staining and hydroxyproline assays showed that among EtOH-fed mice, mice treated with YJ had less fibrosis than mice without YJ (Fig. 8f bottom panel and g). Significant differences between pair-fed mice treated with YJ and pair-fed mice treated with vehicle were rarely identified (Fig. 8). Therefore, these results confirm that peptide YJ, which mimics TSG-6, successfully abolishes CD44 activity and ameliorates alcohol-induced liver fibrosis.

## Discussion

ALD is one of the major causes of preventable morbidity and mortality worldwide and is accompanied by extensive liver fibrosis [1]. However, despite the significant disease burden of alcohol-induced hepatic fibrosis, there are currently no treatments that can prevent or reverse fibrosis [46, 47]. In the absence of effective drug treatments, stem cell-derived factors have emerged as attractive candidates for ALD [48, 49]. We previously demonstrated that TSG-6, which is a cytokine released from MSCs, had therapeutic effects in an animal model of NASH and of hepatic fibrosis [15–17]. Based on this promising therapeutic potential of TSG-6 against liver disease, we studied the effects of TSG-6 in ALD in this research. To study alcohol-induced hepatic damage with fibrosis, various animal models mimicking human-like ALD have been developed and used [50, 51]. Among them, EtOH-containing liquid diets are widely used. The NIAAA model is established by 10 days of ad libitum feeding plus a single binge of ethanol feeding and leads to robust hepatic neutrophil infiltration, but fibrosis is not severe in this model [29]. Hence, the NIAAA model is applicable for exploring the pathological mechanism of alcohol-induced inflammation rather than liver fibrosis [29, 52]. The Lieber–DeCarli model, one of the most widely used ALD models, feeds rodents the EtOH-containing Lieber–DeCarli liquid diet for 4–12 weeks [53]. This model has been applied in numerous studies since 1965, when it was first introduced by Lieber et al. and is considered the appropriate model designed specifically for studying the in vivo effect of alcohol consumption [54]. The Lieber–DeCarli model induces a broad spectrum of hepatic damage, such as macrovesicular steatosis, lobular inflammation, hepatocyte ballooning, and fibrosis [55]. In addition, experimental animals naturally consume a Lieber–DeCarli diet without aversion to alcohol consumption, and there is no need to forcefully feed rodents by gastric injections or surgical intragastric cannulation [29, 52–56]. Therefore, the diet has the advantage of reducing the stress that animals experience during the feeding period. Given that experimental mice should experience stress caused by repeated i.p. injections of TSG-6 or peptide YJ, a less painful model of chronic alcohol consumption was considered most appropriate for the present studies to reveal TSG-6 action in ALD with fibrosis. A long-term ethanol-containing Lieber–DeCarli diet induced severe hepatic injury, such as increased TG accumulation, hepatocyte death, inflammation and fibrosis, successfully modeling ALD (Supplementary Fig. 2, 3 and 4). TSG-6 administration effectively decreased ethanol-induced hepatic damage in mice with ALD (Fig. 1). In particular, hepatic fibrosis and HSC activation were greatly attenuated in TSG-6-treated mice compared with vehicle-treated mice in the EtOH groups (Fig. 2). These results indicate that TSG-6 alleviates alcohol-induced liver fibrosis and HSC activation.

CD44 is a transmembrane protein that regulates multiple cellular processes, such as cell proliferation, migration, and differentiation [18, 19]. In the liver, CD44 was shown to be expressed by infiltrating lymphocytes, Kupffer cells and activated HSCs, and its expression was highly upregulated in fibrotic liver compared with healthy liver [24]. In line with the increase in CD44 levels, soluble CD44 was upregulated in severe acute or chronic liver disease, including hepatitis and cirrhosis [39]. It has also been reported that CD44ICD in the nucleus binds to the CD44ICD response element on the NOTCH1 promoter region and initiates the transcription of Notch1, contributing to liver fibrosis [27]. However, direct evidence proving the production and nuclear localization of CD44ICD to promote HSC activation and hepatic fibrosis was not provided in the research. In addition, except for this article, there has been no paper on CD44ICD in liver fibrosis to date. In the current study, we showed that the elevated expression of CD44ICD by EtOH feeding was significantly alleviated by TSG-6 treatment (Fig. 3c). TSG-6 significantly decreased the level of active nuclear CD44ICD in EtOH-fed mice (Fig. 3d). In parallel with the in vivo data, TSG-6-treated human pHSCs had significantly lower levels of CD44ICD in both the whole lysate and nuclear fraction (Fig. 4). These results suggest that the antifibrotic action of TSG-6 in ALD is mediated by inhibiting the production and/or nuclear translocation of CD44ICD. However, it remains unclear how TSG-6 regulates the activity of CD44ICD. Additional TSG-6-affected factors responsible for CD44 cleavage seem to be required to address the question, although TSG-6 was shown to bind to CD44. Hence, we analyzed the co-IP of TSG-6 using LC‒MS/MS and found that the highly abundant MMP14 in LC‒MS/MS was predicted to have a high possibility of interaction with TSG-6. MMP14 is a membrane-associated matrix metalloproteinase with proteolytic activity and is known to trigger cleavage of the CD44 ectodomain, an essential prerequisite for CD44ICD generation [57]. We found that MMP14 was significantly upregulated in activated HSCs and the livers of EtOH-fed mice with fibrosis as an increase in CD44ICD in these cells and animals (Supplementary Fig. 6). TSG-6 treatment notably decreased both MMP14 and CD44ICD in vivo and in vitro (Figs. 3 and 4 and Supplementary Fig. 6). Based on these results, it was assumed that the interaction of TSG-6 with both MMP14 and CD44 intervened the binding between MMP14 and CD44 and thereby inhibited CD44 cleavage by MMP14. The computational molecular docking program HADDOCK predicted that TSG-6 physically inhibited the catalytic activity of MMP14 and protected CD44 cleavage to CD44ICD (Fig. 5f), and the TSG-6-based peptide YJ designed to interact with the catalytic region of MMP14 successfully suppressed CD44 cleavage, followed by a reduction in CD44ICD production and nuclear localization (Fig. 6). Therefore, these findings explain how TSG-6 attenuates liver fibrosis and HSC activation in ALD mice by showing that TSG-6 directly binds to MMP14 and CD44 and inhibits CD44 activation by blocking MMP14.

In the stable complex of TSG-6, CD44, and MMP14 predicted by HADDOCK, TSG-6 makes polar contacts with the conserved amino acids Glu^240^ and His^249^ of the zinc-binding motif ^239^HEXXHXXGXXH^249^ of MMP14 (Fig. 5f) [58, 59]. In detail, Arg^8^ and Lys^20^ of TSG-6 contact His^249^ and Glu^240^ of MMP14, respectively. The Arg and Lys residues of TSG-6 have been proven to be essential residues for the ligand binding of TSG-6 [60]. These results support that the molecular docking analysis of TSG-6 and MMP14 was successful. Based on these findings, we designed peptides composed of TSG-6 amino acid segments that interact with CD44 and MMP14. (Supplementary Fig. 7b). TSG-6-mimicking peptides were analyzed using the same docking methodology adopted in the analysis of TSG-6, and peptide YJ was shown to block the proteolytic site of MMP14 by directly interacting with the site, similar to TSG-6 (Fig. 6a). The synthesized peptide YJ successfully mimics TSG-6 action, preventing CD44 cleavage by obstructing MMP14 activity in HSCs and alleviating hepatic fibrosis.

Although TSG-6 or the TSG-6-based peptide YJ reduced hepatic expression of full-length CD44 in the livers of EtOH diet-fed mice, they did not impact the protein level of full-length CD44 in HSCs (Figs. 3c, 4a, 6e and 8b). The different expression of CD44 in vivo and in vitro indicates that TSG-6 may influence other types of cells expressing CD44 in addition to HSCs. CD44 was reported to be expressed by liver macrophages and infiltrating lymphocytes [19]. Patouraux et al. [26] demonstrated that CD44 expression was strongly correlated with hepatic recruitment of macrophages in NASH patients. Given the significant alleviation of inflammation, specifically F4/80- or CD68-positive cells, by TSG-6 in our data, a decreased number of these cells could contribute to the reduction in CD44 in vivo. The effect of TSG-6 in macrophages has been reported by several groups. It was reported that TSG-6 treatment induced M2 polarization of macrophages by activating STAT3 signaling in mice fed a chronic-binge Lieber–DeCarli diet for 10 days [61]. Choi et al. [62] reported that TSG-6 blocked TLR2-mediated nuclear translocation of NF-κB in macrophages by interacting with CD44 in these cells. It was also shown that TSG-6 treatment reduced CD44 expression in alloreactive lymphocytes in vitro to suppress lymphocyte activation. Thus, it is possible that TSG-6 treatment induces a decrease in CD44-positive macrophage recruitment into the liver, leading to lowered inflammation and CD44 expression in the livers of EtOH-fed mice.

Previously, our group showed that the interaction of TSG-6 with CD44 activated β-catenin/YAP-1 signaling and reprogrammed activated HSCs into stem-like cells, mitigating HSC activation and liver fibrosis in mice with chronic liver damage from CCl_4_ [28]. The current study also showed alleviation of HSC activation and liver fibrosis by TSG-6 interacting with CD44 in ALD mice. Although different experimental animal models were employed in the two studies, the CD44 level in TSG-6-exposed HSCs was similar to that in vehicle-treated cells in both studies. Given that TSG-6 protects CD44 cleavage from MMP14, intact CD44 maintained by TSG-6 cannot produce CD44ICD to activate HSCs and is also possibly involved in the activation of β-catenin/YAP-1 signaling to switch activated HSCs into stem-like cells, suggesting that TSG-6 has the effect of killing two birds with one stone in reducing HSC activation by maintaining intact CD44. To validate this therapeutic strategy, further studies are needed to reveal whether CD44 maintenance by TSG-6 induces HSC reprogramming by regulating β-catenin/YAP-1 signaling in an ALD model.

## Conclusion

Our results demonstrated that the interaction of TSG-6 with CD44 and MMP14 suppresses CD44 activation by inhibiting CD44 cleavage from MMP14, downregulates CD44ICD, an activator of CD44 signaling, and reduces HSC activation and fibrosis in ALD mice. The TSG-6-based peptide YJ effectively mimics the therapeutic function of TSG-6 and ameliorates hepatic damage, including fibrosis, in EtOH-fed mice. Therefore, our findings suggest that both TSG-6 and peptide YJ regulate pathogenic CD44ICD and have therapeutic potential for treating ALD with fibrosis.

### Supplementary Information

Additional file 1: Supplementary Figure 1. Pharmacokinetic properties of TSG-6 in mice with acute ALD. Supplementary Figure 2. Mice have liver injury with fibrosis at 9 weeks of Lieber–DeCarli alcohol liquid diet. Supplementary Figure 3. Vehicle treatment rarely impacts pathophysiological response of the liver to the diet feeding. Supplementary Figure 4. Liver fibrosis is induced by chronic consumption of EtOH in regardless of vehicle injection. Supplementary Figure 5. Localization of nuclear CD44ICD and α-SMA expression in TSG-6-treated human pHSCs. Supplementary Figure 6. Analysis of MMP14 expression in liver cells and ALD mice. Supplementary Figure 7. Design of peptides mimicking TSG-6 and inhibitory action of the peptide # 4 on HSC activation. Supplementary Figure 8. Localization of nuclear CD44ICD and α-SMA expression in YJ-given human pHSCs. Supplementary Figure 9. TSG-6 rarely impacts CD44ICD production in MMP14-suppressed human pHSCs. Supplementary Figure 10. Peptide YJ downregulated profibrotic genes in mice chronically fed EtOH. Supplementary Table 1. Primer list of qRT-PCR.

## Data Availability

The dataset supporting the conclusions of this article is available in the Mendeley Data repository, https://data.mendeley.com/preview/nj9ky4sfrg?a=7776d9c4-8c25-463d-bfbc-561bd8885da2. The raw LC–MS/MS data required to reproduce these findings are available to download from ProteomeXchange Consortium via the PRIDE partner repository with the dataset identifier PXD047449.

## Abbreviations

ALD: Alcohol-related liver disease
TSG-6: Tumor necrosis factor-inducible gene 6 protein
CD44: Cluster of differentiation 44
ICD: Intracellular domain
ECM: Extracellular matrix
HSCs: Hepatic stellate cells
MSCs: Mesenchymal stem cells
NASH: Nonalcoholic steatohepatitis
H&E: Hematoxylin and eosin
ORO: Oil red O
Col1α1: Collagen 1 alpha 1
α-Sma: Alpha-smooth muscle actin
pHSCs: Primary HSCs
co-IP: Coimmunoprecipitation
LC–MS/MS: Liquid chromatography tandem mass spectrometry
GO: Gene Ontology
MMP14: Matrix metalloproteinase 14
HADDOCK: High ambiguity driven protein‒protein docking
PK: Pharmacokinetics
