# Supplementary material for: Tumor necrosis factor-inducible gene 6 protein and its derived peptide ameliorate liver fibrosis by repressing CD44 activation in mice with alcohol-related liver disease
Source: J Biomed Sci. 2024 May 24;31:54. doi: 10.1186/s12929-024-01042-5 (PMC11127441; doi:10.1186/s12929-024-01042-5)
Supplement: Supplementary file 1 — Additional file 1: Supplementary Figure 1. Pharmacokinetic properties of TSG-6 in mice with acute ALD. Supplementary Figure 2. Mice have liver injury with fibrosis at 9 weeks of Lieber–DeCarli alcohol liquid diet. Supplementary Figure 3. Vehicle treatment rarely impacts pathophysiological response of the liver to the diet feeding. Supplementary Figure 4. Liver fibrosis is induced by chronic consumption of EtOH in regardless of vehicle injection. Supplementary Figure 5. Localization of nuclear CD44ICD and α-SMA expression in TSG-6-treated human pHSCs. Supplementary Figure 6. Analysis of MMP14 expression in liver cells and ALD mice. Supplementary Figure 7. Design of peptides mimicking TSG-6 and inhibitory action of the peptide # 4 on HSC activation. Supplementary Figure 8. Localization of nuclear CD44ICD and α-SMA expression in YJ-given human pHSCs. Supplementary Figure 9. TSG-6 rarely impacts CD44ICD production in MMP14-suppressed human pHSCs. Supplementary Figure 10. Peptide YJ downregulated profibrotic genes in mice chronically fed EtOH. Supplementary Table 1. Primer list of qRT-PCR. [file 12929_2024_1042_MOESM1_ESM.docx]

Additional file for

Tumor necrosis factor-inducible gene 6 protein and its derived peptide ameliorate liver fibrosis by repressing CD44 activation in mice with alcoholic liver disease

Additional file 1 includes:

Supplementary Figure 1. Pharmacokinetic properties of TSG-6 in mice with acute ALD.

Supplementary Figure 2. Mice have liver injury with fibrosis at 9 weeks of Lieber–DeCarli alcohol liquid diet.

Supplementary Figure 3. Vehicle treatment rarely impacts pathophysiological response of the liver to the diet feeding.

Supplementary Figure 4. Liver fibrosis is induced by chronic consumption of EtOH in regardless of vehicle injection.

Supplementary Figure 5. Localization of nuclear CD44ICD and α-SMA expression in TSG-6-treated human pHSCs.

Supplementary Figure 6. Analysis of MMP14 expression in liver cells and ALD mice.

Supplementary Figure 7. Design of peptides mimicking TSG-6 and inhibitory action of the peptide # 4 on HSC activation.

Supplementary Figure 8. Localization of nuclear CD44ICD and α-SMA expression in YJ-given human pHSCs.

Supplementary Figure 9. TSG-6 rarely impacts CD44ICD production in MMP14-suppressed human pHSCs.

Supplementary Figure 10. Peptide YJ downregulated profibrotic genes in mice chronically fed EtOH.

Supplementary Table 1. Primer list of qRT-PCR.


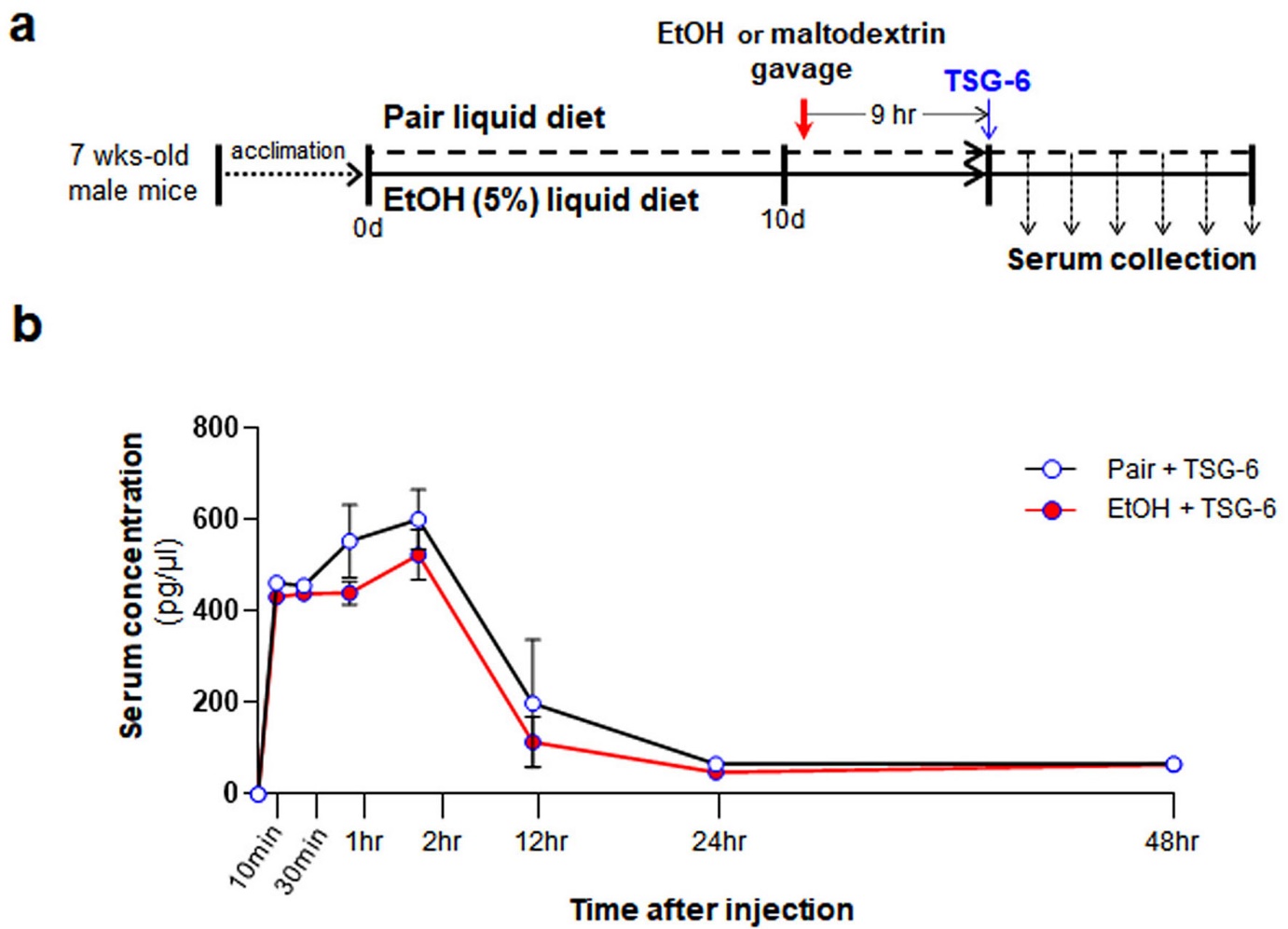


Supplementary Figure 1. Pharmacokinetic properties of TSG-6 in mice with acute ALD.

(a) A scheme for animal experiment to analyze in vivo pharmacokinetics of TSG-6. 7-week-old male C57BL/6 mice were acclimatized for a week and fed either pair or EtOH diet for 10 days. And then these mice were treated with a single dose gavage of maltodextrin solution or ethanol. Nine hours after the gavage feeding, human recombinant TSG-6 was intraperitoneally given to these mice. Jugular vein blood was collected from at 10min, 30min, 1hr, 2hr, 12hr, 24hr and 48hr after TSG6 administration. (b) Pharmacokinetic profile of human TSG-6 in mouse serum of Pair+TSG-6 (n=5) and EtOH+TSG-6 group (n=6). Data are presented as the mean ± S.E.M..

**
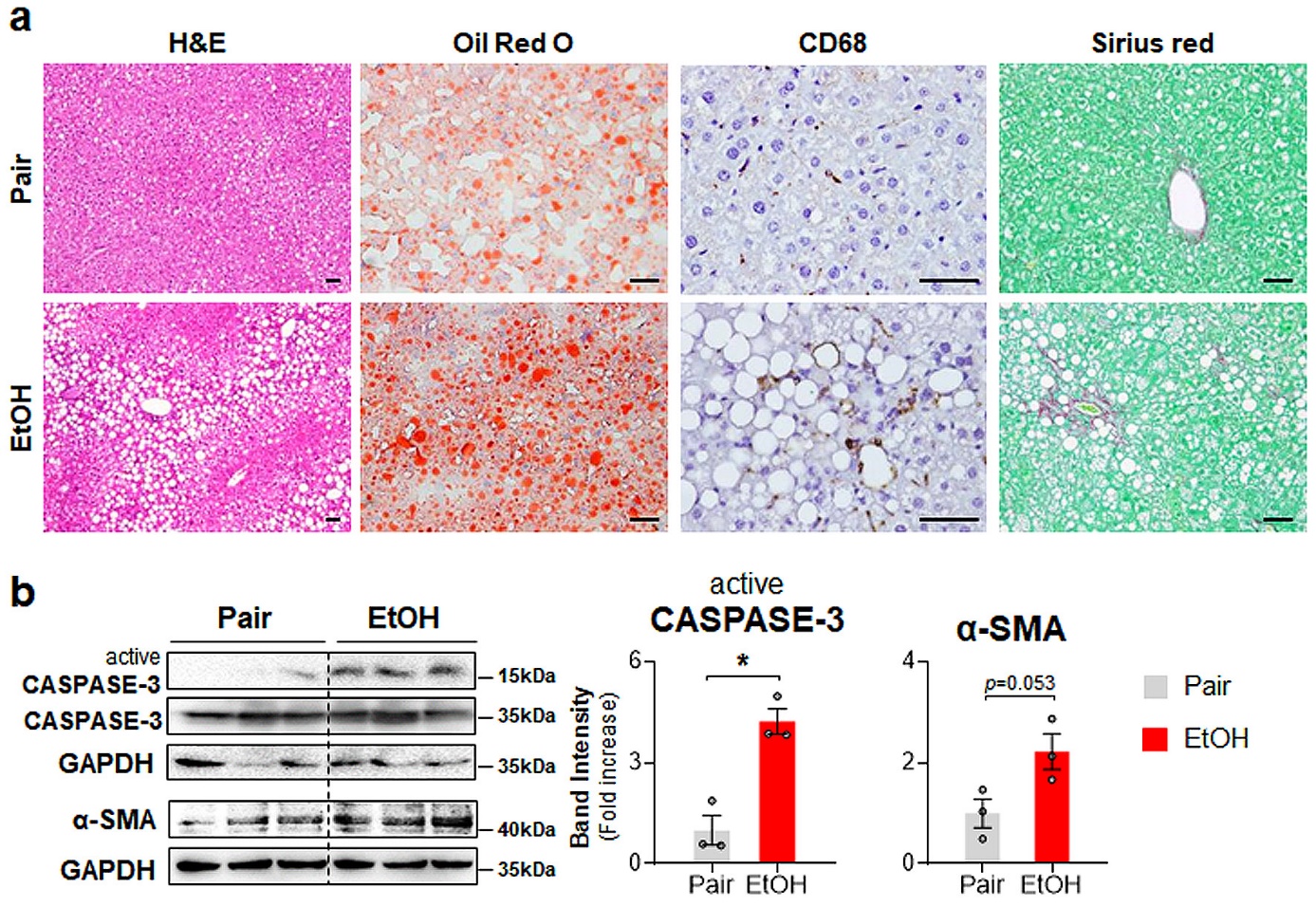
**

**Supplementary Figure 2. Mice have liver injury with fibrosis at 9 weeks of Lieber–DeCarli alcohol liquid diet.**

(a) Representative images of H&E-(first column), Oil-red O-(second column), CD68-(third column) and Sirius Red-stained (fourth column) liver sections from mice fed either isocaloric-containing liquid pair diet or 5% EtOH-having liquid diet for 9 weeks (scale bar, 50 μm). (b) Western blot and cumulative densitometric analyses of active CASPASE-3, total CASPASE-3 and a-smooth muscle actin (a-SMA) in these mice. GADPH were used as internal control. Band densities of active CASPASE-3 were normalized to the expression level of total CASPASE-3 and densities of a-SMA were normalized to the expression level of GAPDH. These data shown represent one of three experiments with similar results from at least three representative mice per each group and are presented as the mean ± S.E.M. (**p*<0.05). Gray circles represent individual data points.


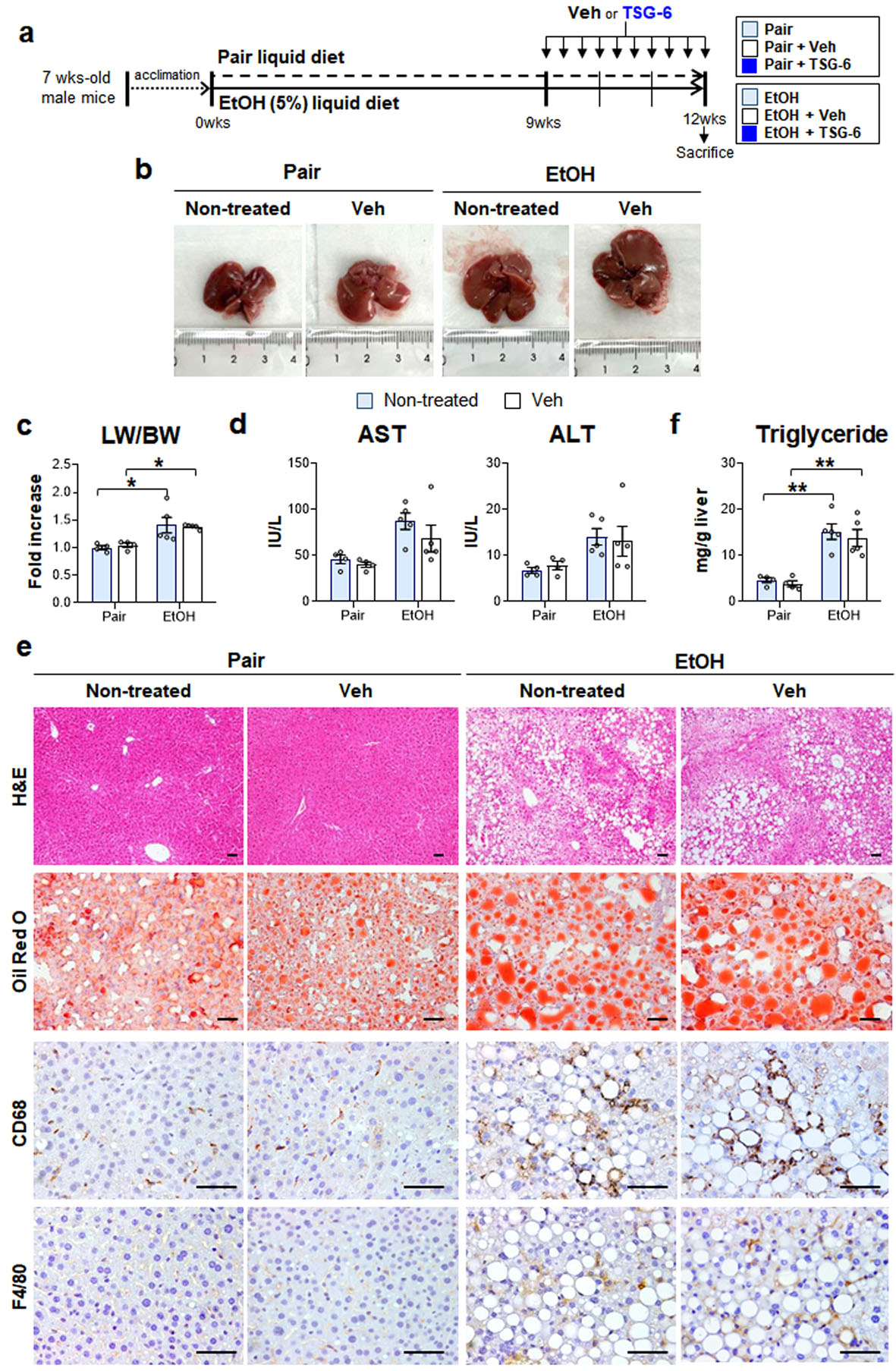


**Supplementary Figure 3. Vehicle treatment rarely impacts pathophysiological response of the liver to the diet feeding.**

(a) A scheme for animal experiment. 7-week-old male C57BL/6 mice which were acclimatized for a week were treated with either pair or EtOH diet for 9 weeks and additionally fed these diets for 3 weeks in parallel with i.p injection of either PBS as a vehicle or TSG-6. (b) Representative macroscopic appearance of livers from the diet-treated mice with or without (non-treated) vehicle. (c) The ratio of liver weight to body weight (LW/BW) and (d) the serum level of AST and ALT of mice from each group. (e) Representative images of H&E (top panel), oil-red O (second panel), CD68-(third panel) and F4/80-stained (bottom panel) liver sections from each group (Scale bar, 50 μm). (f) Hepatic triglycerides (TG) amount of mice from each group. These data shown represent one of three experiments with similar results from at least four representative mice per each group and are presented as the ± S.E.M. (**p*<0.05, ***p*<0.005). Gray circles represent individual data points.


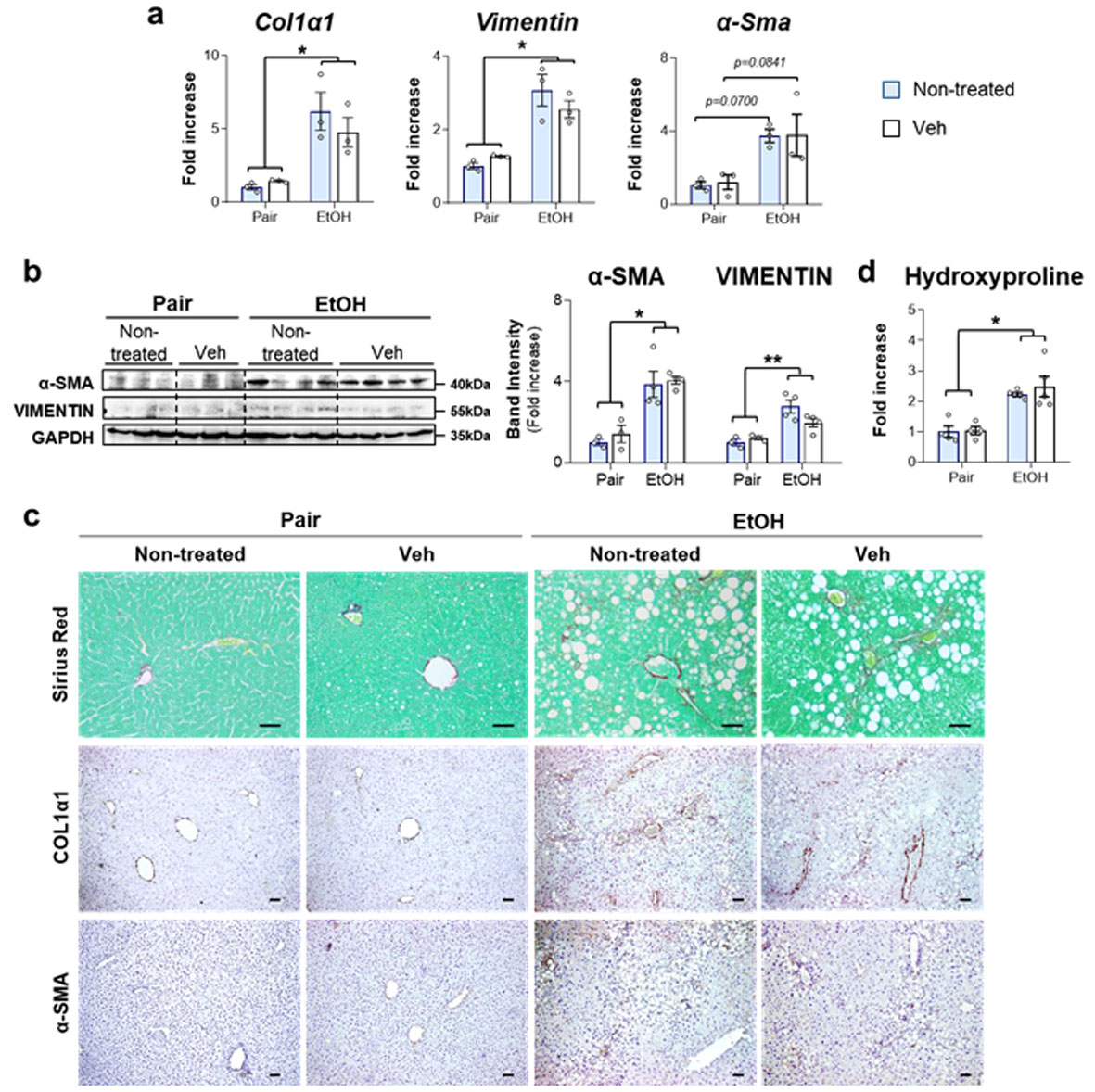


**Supplementary Figure 4. Liver fibrosis is induced by chronic consumption of EtOH in regardless of vehicle injection.**

(a) qRT-PCR analysis of hepatic *collagen type 1 α 1 (Col1α1)*, *Vimentin* and *α-Sma* in representative mice which were exposed to pair- or EtOH diet with or without (non-treated) vehicle. (b) Western blot and cumulative densitometric analysis of α-SMA and VIMENTIN in the liver tissues from representative mice per each group. Band densities were normalized to the expression level of GAPDH, which was used as an internal control. (c) Representative images of Sirius red- (top panel) and COL1α1- (middle panel) and α-SMA-stained (bottom panel) liver sections from these mice (Scale bar, 50 μm). (d) Hepatic hydroxyproline content in liver tissues from representative mice per each group. These data shown represent one of three experiments with similar results from at least four representative mice per each group, and are presented as the ± S.E.M. (**p*<0.05, ***p*<0.005). Gray circles represent individual data points.


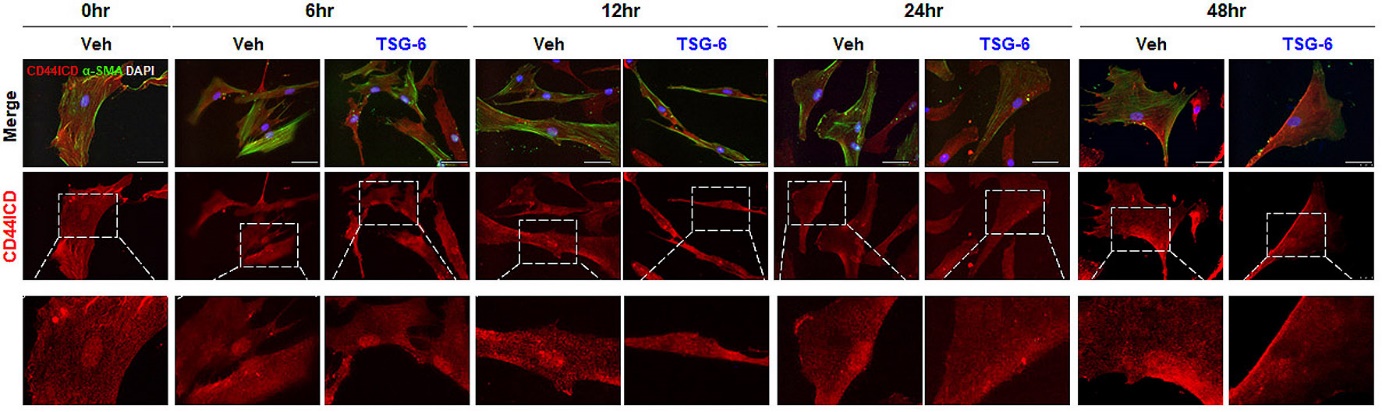


Supplementary Figure 5. Localization of nuclear CD44ICD and expression of α-SMA in TSG-6-treated human pHSCs.

Representative images of double immunofluorescence staining for CD44ICD (red) and α-SMA (green) in human pHSCs after TSG-6 treatment. Magnified images in bottom panel are shown at X60. DAPI (blue) was used as nuclear counterstaining (Scale bar, 50 μm).


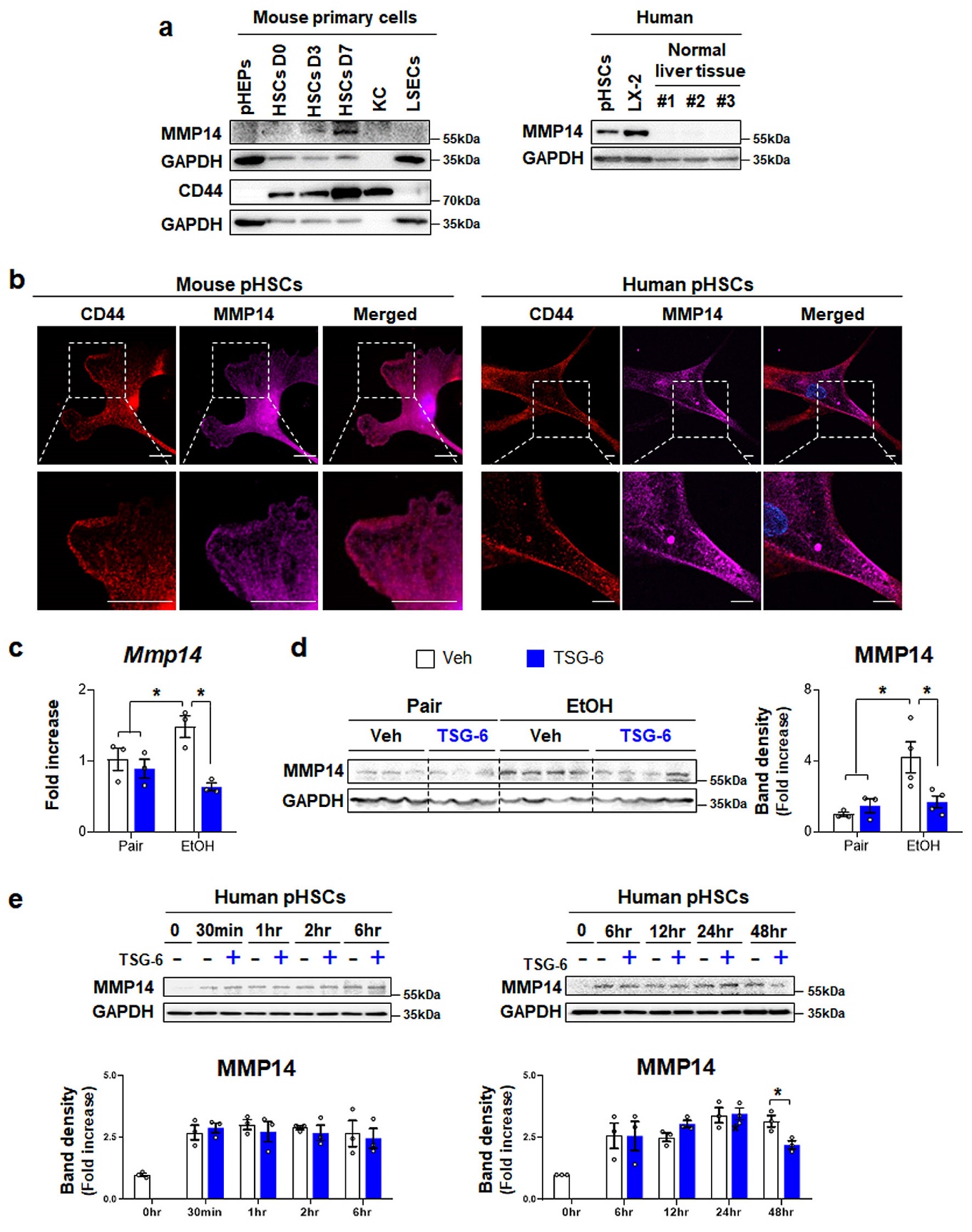


**Supplementary Figure 6. Analysis of MMP14 expression in liver cells and ALD mice.**

(a) Western blot analysis of MMP14 and CD44 in primary hepatocytes (pHEPs), quiescent HSCs (D0 after isolation), culture-activated HSCs (D3 and D7), Kupffer cells and liver sinusoidal endothelial cells (LSEC) which were isolated from WT mice (n≥ 2 /group), and human pHSCs, LX-2 (human HSC line) and healthy liver tissue (n=3). GAPDH was used as internal control. Data shown represent one of the three experiments with similar results. (b) Representative images of double immunofluorescence staining for CD44 (red) and MMP14 (magenta) in mouse and human pHSCs. DAPI (blue) was used as nuclear counterstaining. (Scale bar, 10 μm) (c) qRT-PCR, (d) Western blot and cumulative densitometric analysis of hepatic MMP14 in representative mice which received pair or EtOH diet with either vehicle or TSG-6. GAPDH was used as internal control. Band densities were normalized to the expression level of GAPDH, which was used as an internal control. These data shown represent one of three experiments with similar results from at least four representative mice per each group, and are presented as the ± S.E.M. (**p*<0.05, ***p*<0.005). (e) Western blot and cumulative densitometric analysis of MMP14 in human pHSCs treated with TSG-6. Band densities were normalized to the expression level of GAPDH, which was used as an internal control. Data shown represent one of three experiments with similar results and are presented as the mean ± S.E.M. (*p<0.05). Gray circles represent individual data points.


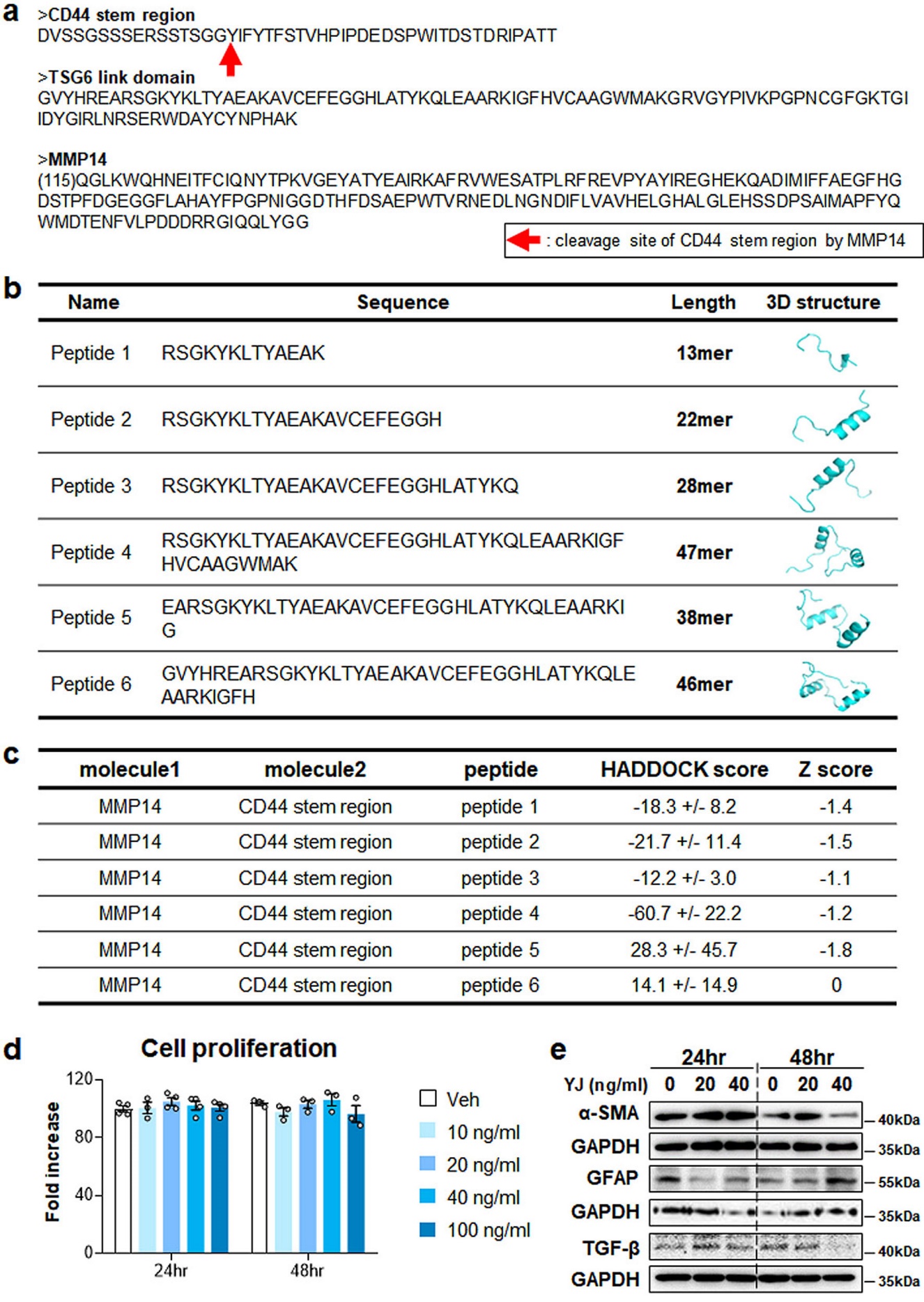


**Supplementary Figure 7. Design of peptides mimicking TSG-6 and inhibitory action of the peptide # 4 on HSC activation.**

(a) Amino acid sequences of CD44 stem region, TSG-6 link domain and MMP14. Red arrow indicates cleavage site of CD44 stem region by MMP14. (b) Amino acid sequence, length, and 3D structure were obtained by alpha-fold of each designed peptide. (c) Results of molecular docking (HADDOCK) analysis of each peptide, CD44 and MMP14. (d) Proliferation of pHSCs exposed to vehicle or the peptide # 4 (YJ) was analyzed by the MTS assay. The mean ± SEM results obtained from three identical experiments are plotted. Gray circles represent individual data points. (e) Western blot analysis for α-SMA, GFAP and TGF-β in pHSCs treated with 20 or 40ng/ml of peptide YJ for 24 and 48 hours. GADPH were used as internal control. Data shown represent one of three experiments with similar results.


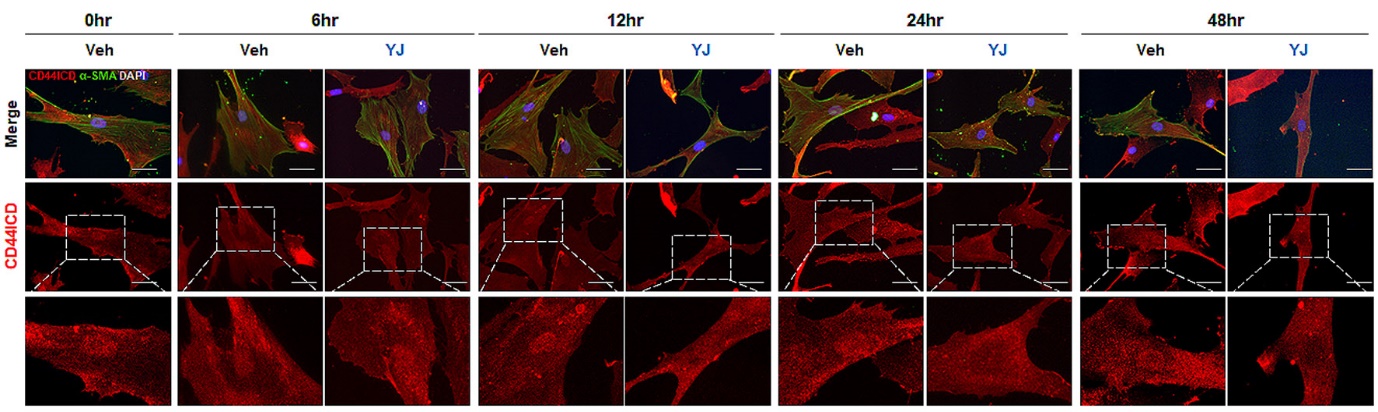


Supplementary Figure 8. Localization of nuclear CD44ICD and expression of α-SMA in YJ-given human pHSCs.

Representative images of double immunofluorescence staining for CD44ICD (red) and α-SMA (green) in human pHSCs after YJ treatment. Magnified images in bottom panel are shown at X60. DAPI (blue) was used as nuclear counterstaining (Scale bar, 50 μm).


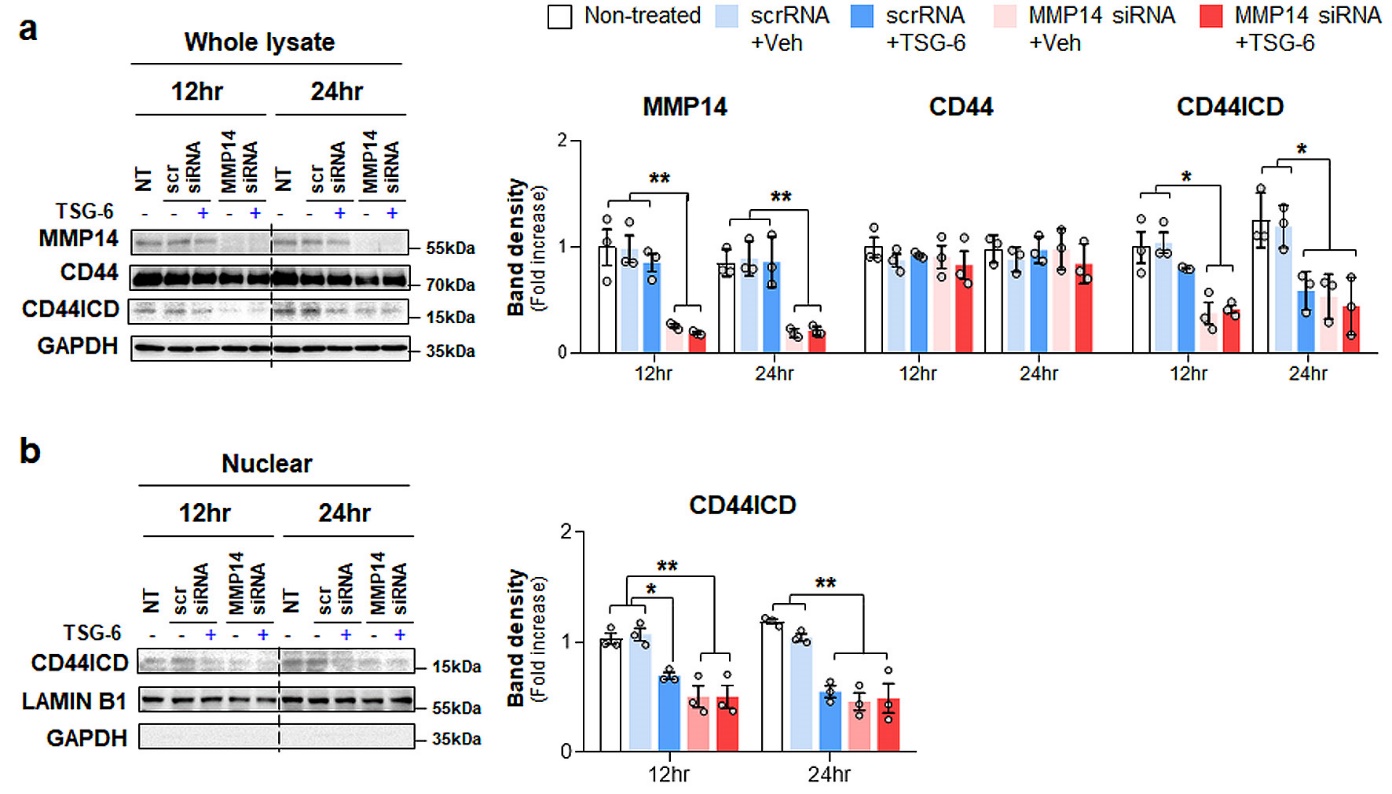


Supplementary Figure 9. TSG-6 rarely impacts CD44ICD production in MMP14-suppressed human pHSCs.

(a) Western blot and cumulative densitometric analysis of MMP14, CD44 and CD44ICD in whole lysate of TSG-6- or vehicle-treated human pHSCs transfected with scramble siRNA (scrRNA) or siRNA targeting MMP14 (MMP14 siRNA). pHSCs were treated with scrRNA or MMP14 siRNA for 24 hours and then exposed to vehicles or TSG-6 for 12 or 24 hours. GAPDH was used as an internal control. (b) Western blot and cumulative densitometric analysis of nuclear CD44ICD in these cells. LAMIN B1 and GAPDH was used for positive and negative internal control, respectively. Band densities were normalized to the expression level of internal control. Data shown represent one of three experiments with similar results and are presented as the mean ± S.E.M. (*p<0.05, **p<0.005). Gray circles represent individual data points.


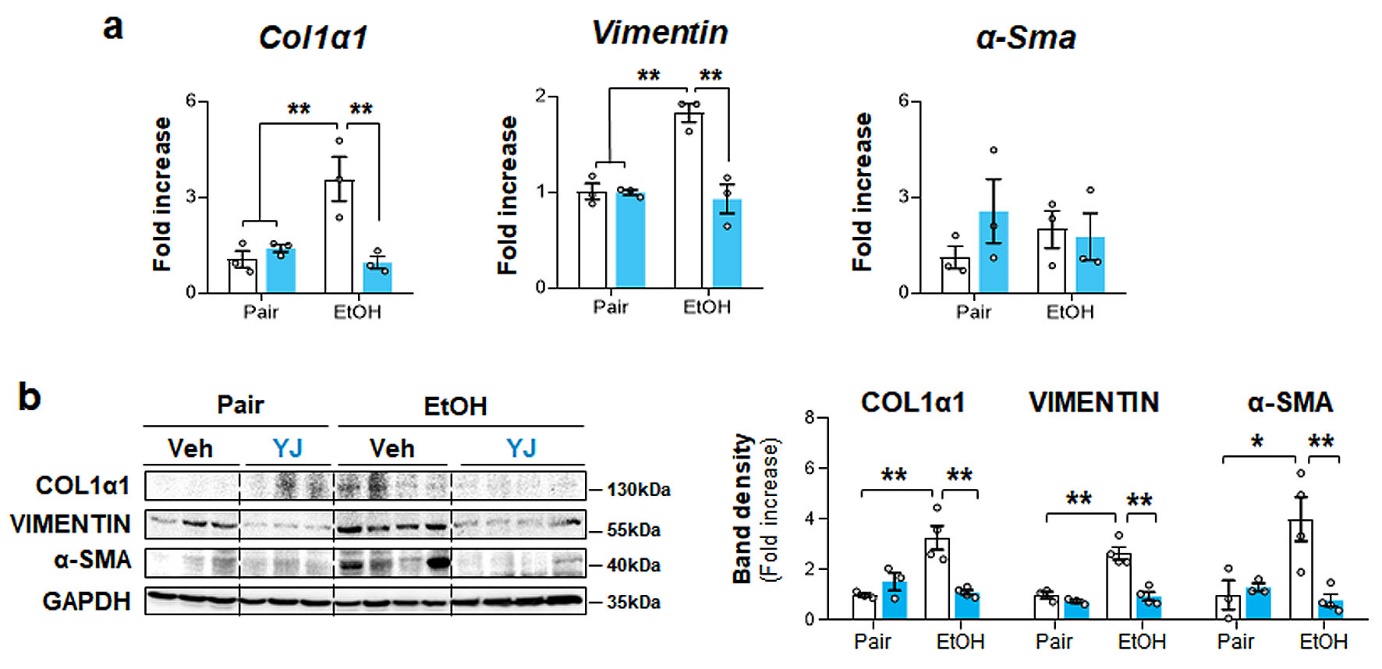


**Supplementary Figure 10. Peptide YJ downregulated profibrotic genes in mice chronically fed EtOH.**

(a) qRT-PCR analysis of *Col1α1,* *Vimentin* and *α-Sma* in vehicle or peptide YJ-treated mice fed pair or EtOH diet. (b) Western blot and cumulative analysis of COL1α1, α-SMA and VIMENTIN in the liver tissues from each group. Band densities were normalized to the expression level of internal control. These data shown represent one of three experiments with similar results from at least four representative mice per each group, and are presented as the mean ± S.E.M. (**p*<0.05, ***p*<0.005). Gray circles represent individual data points.

**Supplementary Table 1. Primer list of qRT-PCR.**

| Human | | |
| --- | --- | --- |
| Gene | Forward sequence | Reverse sequence |
| *TGF-β* | TTGACTGAGTTGCGATAATGTT | GGGAAATTGCTCGACGAT |
| *α-SMA* | GTGACGAAGCACAGAGCAAA | CTTTTCCATGTCGTCCCAGT |
| *COL1α1* | CAGATCACGTCATCGCACAA | TGTGAGGCCACGCATGAG |
| *TIMP1* | ACTTCCACAGGTCCCACAAC | CACTGTGCATTCCTCACAGC |
| *CTGF* | TCCCAAAATCTCCAAGCCTA | GTAATGGCAGGCACAGGTCT |
| *9S* | GACTCCGGAACAAACGTGAGGT | CTTCATCTTGCCCTCGTCCA |
| Mouse | | |
| Gene | Forward sequence | Reverse sequence |
| *Col1α1* | GAGCGGAGAGTACTGGATCG | GCTTCTTTTCCTTGGGGTTC |
| *α-Sma* | AAACAGGAATACGACGAAG | CAGGAATGATTTGGAAAGGA |
| *Vimentin* | GCAGGGCATCGTTGTTC | GCTTCTCTGGCACGTCTTGA |
| *Mmp14* | TGCCCAATGGAAAGACCTAC | TGCCCATGAATGACCCTCT |
| *9s* | GGGCCTGAAGATTGAGGATT | CGGGCATGGTGAATAGATTT |

These primer sequences were used for qRT-PCR. The expression level of mRNA was normalized by the expression level of 9S mRNA.
